# DNA Condensation by Peptide-Conjugated PAMAM Dendrimers. Influence of Peptide Charge

**DOI:** 10.1021/acsomega.3c05140

**Published:** 2023-11-15

**Authors:** Corinna Dannert, Ingrid Mardal, Rahmi Lale, Bjørn Torger Stokke, Rita S. Dias

**Affiliations:** † Biophysics and Medical Technology, Department of Physics, NTNU—Norwegian University of Science and Technology, Trondheim N-7491, Norway; ‡ Department of Biotechnology and Food Science, NTNU—Norwegian University of Science and Technology, Trondheim N-7491, Norway

## Abstract

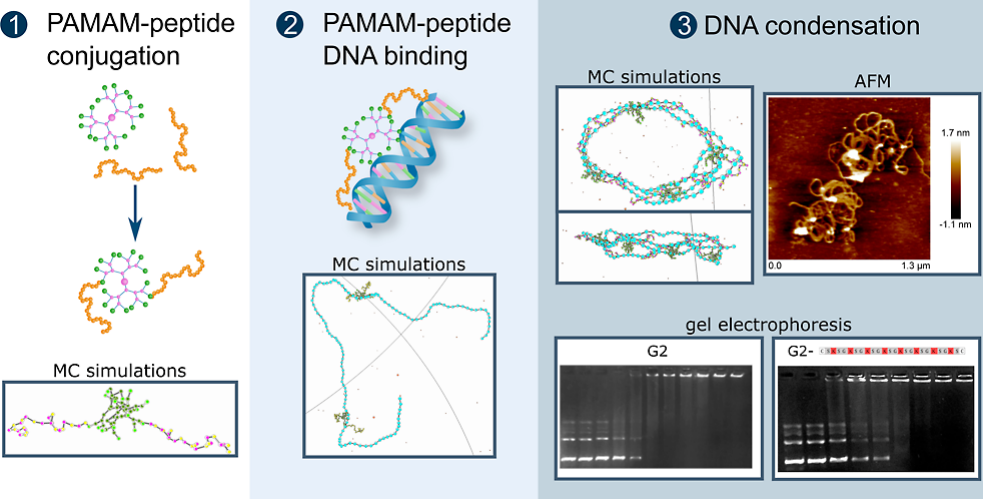

Nucleic acid delivery to cells is an important therapeutic strategy that requires the transport of nucleic acids to intracellular compartments and their protection from enzymatic degradation. This can be achieved through the complexation of the nucleic acids with polycations. Poly(amidoamine) (PAMAM) dendrimers and peptide-conjugated dendrimers have been investigated as delivery vectors. Inspired by these studies and the role of flexible peptide domains in protein–DNA interactions, we studied the impact of conjugating two peptides (tails) to generation 2 (G2) PAMAM dendrimers on DNA condensation and polyplex formation. Using gel electrophoresis, dye exclusion assays, atomic force microscopy, and Monte Carlo simulations, it is shown that the steric impact of neutral peptide tails is to hinder the formation of DNA-G2 polyplexes composed of multiple DNA chains. If the tails are negatively charged, which results in overall neutral G2 conjugates, then the interaction of G2 with DNA is hindered. Increasing the net positive charge of the tails resulted in the complexation capacity of G2 with the DNA being restored. While DNA complexation is obtained for a similar net charge balance for G2 and G2 conjugates with positive tails, fewer of the latter are required to achieve a comparable condensation degree. Furthermore, it is shown that about 40% of the DNA remains accessible to binding by small molecules. Overall, this shows that tuning the net charge of peptide tails conjugated to PAMAM dendrimers offers a handle to control the complexation capacity of DNA, which can be explored as a novel route for optimization as gene delivery vehicles.

## Introduction

Polynucleotide delivery to target cells has developed to be an important strategy for changing genetic expression, either by introducing nonexisting genetic code or silencing the translation of existing code.^1,2^ Successful realization of this requires the nucleic acid molecules to be protected from enzymatic degradation,^3^ to cross the cell membrane from the extracellular domain to the intracellular compartment (and the nuclear envelope if its mode of action requires so), and finally to be available to the cell machinery.

Poly(amidoamine) (PAMAM) dendrimers are water-soluble molecules that strongly interact with DNA and have thus been investigated as delivery vectors for nucleic acids.^4−10^ The large density of cationic charges in PAMAM dendrimers facilitates their association with nucleic acid chains, which is driven by the entropic gain associated with counterion release, similar to that reported for polycation–polyanion complexation.^11^ The presence of dendrimers along the nucleic acid chains induces attractive interactions within and between the polynucleotides, driven by ion-correlation effects and bridging,^12^ leading to the formation of complexes (polyplexes, often also called dendriplexes) whose characteristic morphologies depend on the generation of the dendrimers,^13^ among other factors. When considering long nucleic acid chains, such complexation reduces the overall dimensions of the nucleic acids, protect them from degradation by nucleases^14^ and, in case of DNA, inhibit its gene transcription.^5^

Reducing the dimensions of the polynucleotides facilitates the endocytosis of the polyplexes, but it is fundamental that once in the cell, the polyplexes escape the endosomes, thus preventing their exclusion from the cell *via* exocytosis or the digestion of the polynucleotides *via* maturation of the endosomes to lysosomes.^15^ PAMAM dendrimers are believed to facilitate endosomal escape, in a mechanism initially attributed to the ability of the dendrimer to buffer the acidification of the endosome,^4^ leading to its swelling and rupture (proton sponge theory).^16,17^ Recently, other theories that do not involve the rupture of the endosomes have been put forward, namely, the polyplex-mediated and the polymer-mediated membrane disruption hypotheses.^18^

As an additional advantage, the amine groups at the surface of the dendrimers facilitate the attachment of functional groups to PAMAM dendrimers, which can enhance their functional properties. For example, conjugating PAMAM dendrimers to histidine-arginine dipeptides and cholesterol has been shown to further improve the ability to escape the endosome.^19^ In another example, cyclic RGD (Arg-Gly-Asp) peptides have been conjugated to generation 5 (G5) PAMAM dendrimers, with the purpose of mediating siRNA delivery to malignant glioma cells. The dendrimer modifications did not reduce the ability of the dendrimers to form complexes with the siRNA and were found to enhance the delivery of siRNA to three-dimensional multicellular spheroids of tumor cells, possibly mediated by enhanced integrin-mediated delivery.^7^ Also with the aim of improving gene silencing, peptides targeting transferrin and growth factor receptors, overexpressed in a variety of tumor cells, have been conjugated to G5 PAMAM dendrimers *via* a polyethylene glycol (PEG) linker.^20^ The modifications again did not seem to greatly affect the complexation of siRNA to the dendrimers, and the dendriplexes were able to mediate a decrease in the expression of the target gene compared to an unspecific siRNA control. In another work, peptides displaying a high affinity toward mesenchymal stem cells were conjugated to G5 PAMAM dendrimers.^8^ It was found that the peptides hindered to some extent the condensation of DNA, as probed by gel electrophoresis, but the formed complexes displayed a high affinity toward the stem cells. In addition, the functionalized dendrimers displayed significantly lower toxicity than the native dendrimers, which was attributed to the partial shielding of primary amines that are predominantly responsible for cytotoxicity effects. Other reports of improved gene delivery coupled with lowered toxicity using peptide-modified dendrimers have emerged in recent years.^19,21−23^ Indeed, despite the advantageous properties mentioned above, it is known that PAMAM dendrimers show concentration- and generation-dependent cytotoxic, due to the associated increase in charge of the dendrimer.^9^ In this respect, conjugating PAMAM with several PEG chains has been reported to increase their biocompatibility.^24,25^ On the other hand, it has been reported that PEG can, in rare cases, lead to unwanted side effects, such as allergic reactions^26,27^ and the PEG-induced shielding effect can decrease the ability of the complexes to efficiently bind and condense DNA.^20^

With the aim of decreasing the toxicity of the PAMAM dendrimers while keeping their ability to condense DNA, we propose, in this work, the use of low-generation PAMAM dendrimers (G2) with conjugated peptides. Contrary to the work described above, where peptides were used with the purpose of targeting specific molecules, here we take inspiration from intrinsically disordered domains in proteins that naturally interact with DNA to tune the interactions between the proteins and DNA. Examples are the histone tails, believed to mediate interactions between nucleosomes in genome packing in eukaryotic cells,^28^ and the p53 protein, whose intrinsically disordered terminus is believed to aid in the search for specific DNA sequences.^29,30^

Furthermore, by systematically changing the overall charge, charge density, and charge distribution of the conjugated peptides (instead of focusing on particular sequences), we aim to evaluate the effect of peptide composition on the ability of the conjugated dendrimers to condense DNA and the impact they have on the structure of the polyplexes. With this in mind, peptides with 24 or 25 amino acids (a.a.) were designed and conjugated to G2 dendrimers.

In addition to peptides containing neutral amino acids and an ampholytic motif, used to assess the steric effects of the tails, anionic and cationic peptides with the charged amino acids organized either in blocks or homogeneously distributed along the peptide chain were studied. The number of charged a.a. was selected to span the range from peptide-induced charge reversal of the G2 dendrimers to doubling the valence of the G2 dendrimers when conjugated with two peptides.

## Materials and Methods

### Materials

PAMAM dendrimer generation 2.0 (G2) with ethylenediamine cores (supplied as 20 wt % in methanol), Pur-A-Lyzer Mega Dialysis Kit with MWCO of 1 kDa, phosphate-buffered saline (PBS) tablets, ethylenediaminetetraacetic acid (EDTA), dithiothreitol (DTT), sulfo-LC-SPDP {sulfosuccinimidyl 6-[3′-(2- pyridyldithio)propionamido]hexanoate}, and agarose were purchased from Sigma-Aldrich/Merck. 6× Tritrack DNA loading dye was purchased from Thermo Fisher Scientific, 10× TAE electrophoresis running buffer was bought from Millipore, and GelStar 10,000× dye was purchased from Lonza. Ultrapure water (resistivity 18.2 MΩ cm, Milli-Q plus, Merck Millipore) was employed in the experiments.

The sequence of the plasmid DNA used in the dye exclusion assays and gel electrophoresis was created using Benchling (see Figure S1 for the map and gene bank file for the sequence), and the 3605 bp long plasmids were ordered from Integrated DNA Technologies (IDT). The DNA was multiplied using a QIAprep Spin Miniprep Kit from QIAGEN, and the sample was diluted in 10 mM Tris-Cl (pH 8.5) buffer. The concentration of DNA was measured by using a Thermo Scientific NanoDrop spectrophotometer. gWiz-Luc plasmid DNA purchased from Aldevron (Fargo, ND) was used in dynamic light scattering (DLS) and atomic force microscopy (AFM) imaging.

Custom-designed peptides were purchased from GenScript. The sequences, presented in the column to the right in Table 1, include cysteine amino acids in the ends for conjugation. The short names given to the peptides (left column in Table 1) indicate the charged nature and the distribution of the a.a., with *N* referring to negatively charged aspartic acid, *P* representing positively charged lysine, and 0 standing for neutral a.a., either glycine, serine, or proline. The stock solutions of peptides were diluted in PBS buffer (0.5 M NaCl and 10% glycerol, pH 7.4).

**Table 1:**
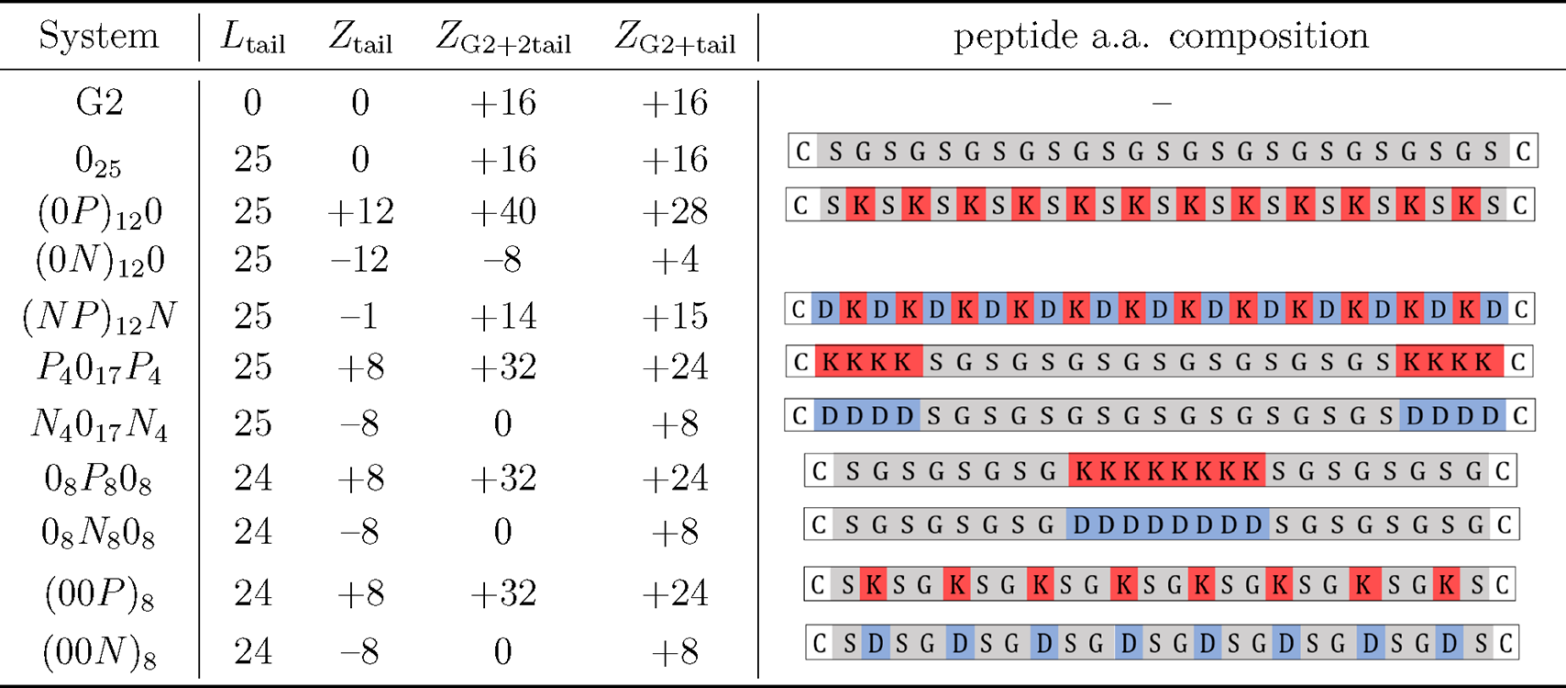
Overview of the Peptide Composition Used in the Experimental Work (Right-Hand-Side Column, with the Colors Highlighting the a.a. Charge) and of the Parameters Varied in the Simulations^a^

a *L*_tail_ is the number of monomers of the tails, whose composition matches those used experimentally and that together defines the overall charge of each tail (*Z*_tail_). *Z*_G2+2tail_ and *Z*_G2+tail_ indicate the overall charge of conjugates consisting of one G2 and two or one peptide tails, respectively. The short names given to the peptides (left column) indicate the a.a. distribution with *N*, *P*, and 0 referring to negative, positive, and neutral a.a., respectively. The additional peptide (0*N*)_12_0 included in the simulations was not studied experimentally.

### PAMAM-Peptide Conjugation

The conjugation of peptides to the PAMAM dendrimers was based on the protocols by Santos *et al.*(8) and Waite and Roth.^7^

**Figure 1:**
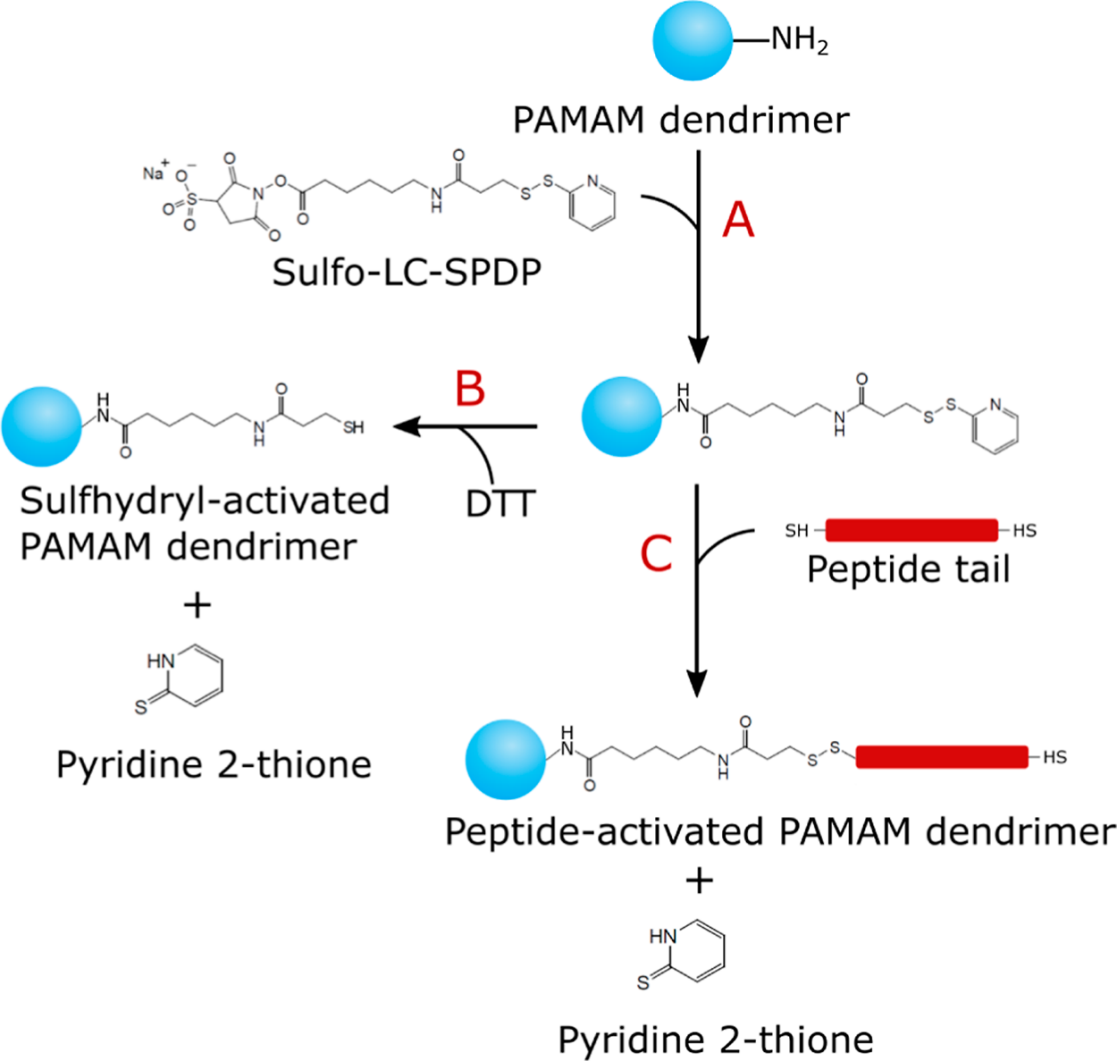
Schematics of conjugation of PAMAM with the peptide tails. Reaction A: attachment of SPDP linker to PAMAM dendrimers. Reaction B: DTT assay to assess the amount of linkers attached to PAMAM. Reaction C: conjugation of peptides to PAMAM-SPDP. Adapted with permission from Santos *et al.*(8) Receptor-Mediated Gene Delivery Using PAMAM Dendrimers Conjugated with Peptides Recognized by Mesenchymal Stem Cells. Molecular Pharmaceutics 2010, 7, 763–774. Copyright 2010 American Chemical Society.

First, the G2 dendrimers were dialyzed against 0.8 L PBS-EDTA (10 mM PBS, 1 mM EDTA, pH 7.4) buffer for 7 h, followed by a buffer exchange, and further dialyzed overnight. The G2 solution was removed from the tubes and diluted to a final concentration of 0.1 mM. The concentration of G2 after the dialysis was assessed based on the weight of the (wet) Pur-A-Lyzer dialysis tubes before and after adding the G2 solutions and after dialysis, assuming that all G2 was contained in the tube during the dialysis. Second, sulfo-LC-SPDP was added to the G2 solution at a molar ratio sulfo-LC-SPDP/G2 of 6:1, and the solution was stirred for 2.5 h at room temperature (Reaction A, Figure 1), to functionalize the G2. Unreacted SPDP was subsequently removed by dialysis overnight against 3 L of PBS-EDTA buffer. Finally, the peptides were conjugated to the G2–SPDP complexes by adding peptides with a molar ratio peptide/SPDP of 1:1 to the G2-SPDP solutions, and the reaction (Reaction C, Figure 1) was allowed to proceed overnight at room temperature.

DTT assays were performed both before (Reaction B in Figure 1) and after the addition of peptides to G2-SPDP, to assess the efficiency of the conjugation. This was realized by adding 10 μL of stock solution of 15 mg/mL DTT in PBS-EDTA buffer to 1 mL of 0.01 mM G2-SPDP and G2-peptide conjugates and left to equilibrate for 15 min at room temperature. The absorbance at 343 nm originating from pyridine-2-thione, a product of the reaction, was determined (Agilent 8453 UV/Vis spectrophotometer) and used as the basis to determine the average number of SPDP conjugated to each G2 and the number of peptides attached to the linkers. With this procedure, the average number of peptides per dendrimer was assessed to be 2.3. See the Supporting Information and Figure S2 for more detailed information on the conjugation and optimization of the procedure. ^1^H NMR was additionally used to assess the conjugation of the (00*P*)_8_ peptide to G2, using D_2_O as the solvent and a 600 MHz Avance III HD NMR spectrometer. For these experiments, SPDP was dissolved in D_2_O, while G2, G2-SPDP, the peptide (00*P*)_8_, and the conjugate G2-(00*P*)_8_ were concentrated using a spin concentrator tube (Amicon Ultra Millipore centrifugal filter, MWCO 3000) and then diluted in D_2_O and transferred to an NMR tube (Wilmad 5 mm NMR tube, Sigma-Aldrich). The proton spectra and their interpretation are presented in the Supporting Information (Figures S3 and S4). In short, we have identified NMR peaks that either disappear or shift, indicating the successful conjugation of the SPDP linker to G2 and the peptide to the linker.

### Dye Exclusion Assays

45 μL of a 4.44 μg/mL DNA solution diluted in PBS-EDTA buffer was mixed with 45 μL of G2 or G2-peptide solutions, both in PBS-EDTA buffer, with varying concentrations and left to equilibrate at room temperature for at least 1 h. The pH of the mixture was 7.4. 10 μL of 100× GelStar was added to each sample and left for another 30 min. The fluorescence intensity of GelStar was determined at an emission wavelength of 540 nm using an excitation wavelength of 493 nm (Spectramax I3X Multimode microplate reader, Molecular Devices).

The results were analyzed as a function of the nominal molar ratio, *r*_molar_, between G2, peptides, or G2 conjugates and DNA, or the nominal electrostatic ratio between the charges on the G2, peptides, or G2 conjugates (*Z*_vector_) and the negative charges on DNA (*Z*_DNA_), *r*_charge_, according to
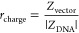
1For the experimental systems, and under the used buffer conditions, we assume that all primary amine end groups of the dendrimers are protonated while the tertiary amino groups are nonprotonated.^31,32^ In addition, 2.3 positive charges were subtracted from the conjugated dendrimers due to the binding of the SPDP linker to and subsequent neutralization of the amine groups. The p*K*_a_ values of aspartic acid (D) and lysine (K) are 3.4 and 10.7, respectively.^33^ So, the assumption that all D and K a.a. are charged at the used pH is reasonable.

### Gel Electrophoresis

For the characterization of the DNA complexes by gel electrophoresis, equal volumes of DNA and vectors were mixed and left to equilibrate at room temperature for at least 1 h. The concentration of DNA was 10 μg/mL in all samples with varying vector concentrations. 10 μL of the samples was mixed with 2 μL of 6× loading dye and transferred to wells in 1% agarose gels in TAE buffer containing 5 μL of 10,000× GelStar. The gels were run for 40 min at 120 V. A Benchtop 3UV transilluminator at 302 nm was used to visualize the bands.

### Dynamic Light Scattering

DLS was used for further characterization of DNA complexation by G2 and G2 conjugates using a Zetasizer Nano ZS from Malvern Panalytical. The experiments were conducted using a scattering angle of 175°, and the apparent hydrodynamic diameter was assessed using the software accompanying the instrument. 1 mL of gWiz-Luc DNA and 8 to 112 μL of different vectors were mixed and left to equilibrate at room temperature for at least 1 h. The concentration of DNA was 10 μg/mL in all samples with varying vector concentrations.

### Atomic Force Microscopy

AFM was conducted by employing a Bruker Multimode Atomic Force Microscope equipped with an E-scanner. The preparation of the dried specimens for AFM followed a procedure involving the deposition of the sample on mica, drying it under a low stream of nitrogen, and subsequently vacuum drying it, as outlined by Maurstad *et al.*(34) In more detail, DNA was diluted in Milli-Q water to 10 μg/mL, mixed with G2 or G2 conjugates at a *r*_charge_ of 2, and left to equilibrate for at least 1 h. An aliquot of the sample was then deposited onto freshly cleaved mica and incubated for 5 min. Excess solution was removed by the gentle application of a nitrogen stream at low pressure, followed by vacuum drying at a pressure of 1.3 mPa or lower for at least 2 h. Silicon nitride cantilevers (PPP-NCH-W, PointProbe Plus, Nanosensors) with nominal spring constants (10–130 N/m) and nominal resonance frequencies (200–500 kHz) were used. The instrument was operated in tapping mode as described previously by Stokke *et al.*(35) The topographs were flattened in the Nanoscope AFM software line-by-line using a third-order polynomial.

### Monte Carlo Simulations

**Figure 2:**
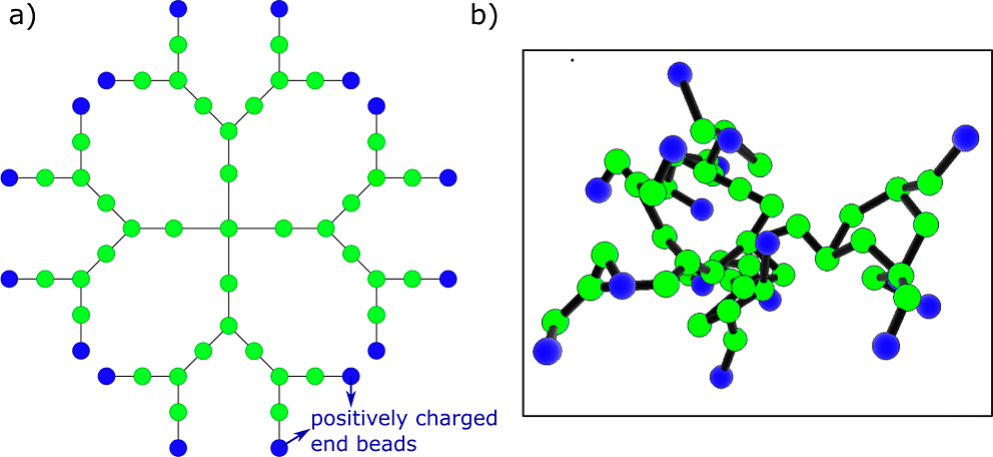
(a) Schematic of the hierarchical G2 PAMAM dendrimer structure used in MC simulations. (b) Snapshot of the G2 model. Inner neutral beads are shown in dark green, and positively charged end groups are shown in light green.

The DNA, peptides, G2, and peptide-conjugated G2 were represented using coarse-grained bead-spring models, derived from the established Kremer–Grest polymer model.^36,37^ The DNA consisted of *N*_mon_^DNA^ = 120 hard spheres (monomers) with a radius of *R*_mon_^DNA^ = 4 Å and a charge of *Z*_mon_^DNA^ = −1. Each a.a. in the peptide chains was described with a hard-sphere with radius *R*_mon_^tail^ = 1.5 Å. The a.a. sequence of the modeled peptides corresponded to those used in the experiments but excluding the cysteine (C) termini (Table 1). More specifically, lysine (K) and aspartic acid (D) were described using hard spheres with *Z* = 1 and *Z* = −1, respectively, while spheres with *Z* = 0 were used to mimic serine (S) and glycine (G). The G2 dendrimers were described as a branched hierarchical structure with four dimers connected to a single central bead. From each of the end beads of the dimers, two more dimers are connected, representing a generation. See Figure 2 for a scheme of how the dendrimers were designed in the simulations. All the beads in the dendrimer were considered to be neutral, with the exception of the 16 end groups that were given a charge of *Z*_mon_^G2^ = 1. The beads of the PAMAM dendrimer had a radius of *R*_mon_^G2^ = 1.5 Å and the overall diameter of the dendrimer was found to be around 15 Å, determined using the radial distribution function between the central beads and the charged end groups of G2. This value was roughly half of the diameter of reported experimental data,^38^ which guaranteed that the proportions between the diameters of the dendrimers and the DNA models were maintained equal to those of the real molecules.

For the peptide-conjugated G2, the peptide chains were attached to the end monomers of G2 by a harmonic potential. In addition to the variation in the composition of the peptide tails (see Table 1), the number of peptide tails per dendrimer was taken to be 1 or 2.

For each charged particle on the DNA, peptides, and G2, a counterion particle was added to the system, with equal but opposite charge and radius *R*_ci_ = 1.2 Å.

Full simulation details and model parameters are given in the Supporting Information. In brief, we simulated one DNA molecule and a varying number of peptides, G2, or peptide-conjugated G2 in a spherical simulation box with radius *R*_cell_ = 1200 Å.

The simulations were performed in the canonical ensemble with a temperature of 298 K using the standard Metropolis Monte Carlo (MC) algorithm,^39^ with random displacements of all individual particles, as well as of the entire (DNA and peptide) chains and G2 within the simulation cell. In addition, pivot moves were also attempted for both linear chains and branched structures. Equilibration and production runs were performed with 2.0 × 10^6^ and 3.0 × 10^6^ MC steps, respectively, and at least six independent runs were conducted for each system. Ensemble averages were calculated from the production runs. The simulation used the MolSim package (version 6.4.7) developed by Reščič Jurij and Linse Per,^40^ with modifications by the authors, and was carried out at the IDUN cluster in NTNU.^41^

## Results and Discussion

### DNA Condensation by Conjugated Dendrimers

#### Gel Electrophoresis

**Figure 3:**
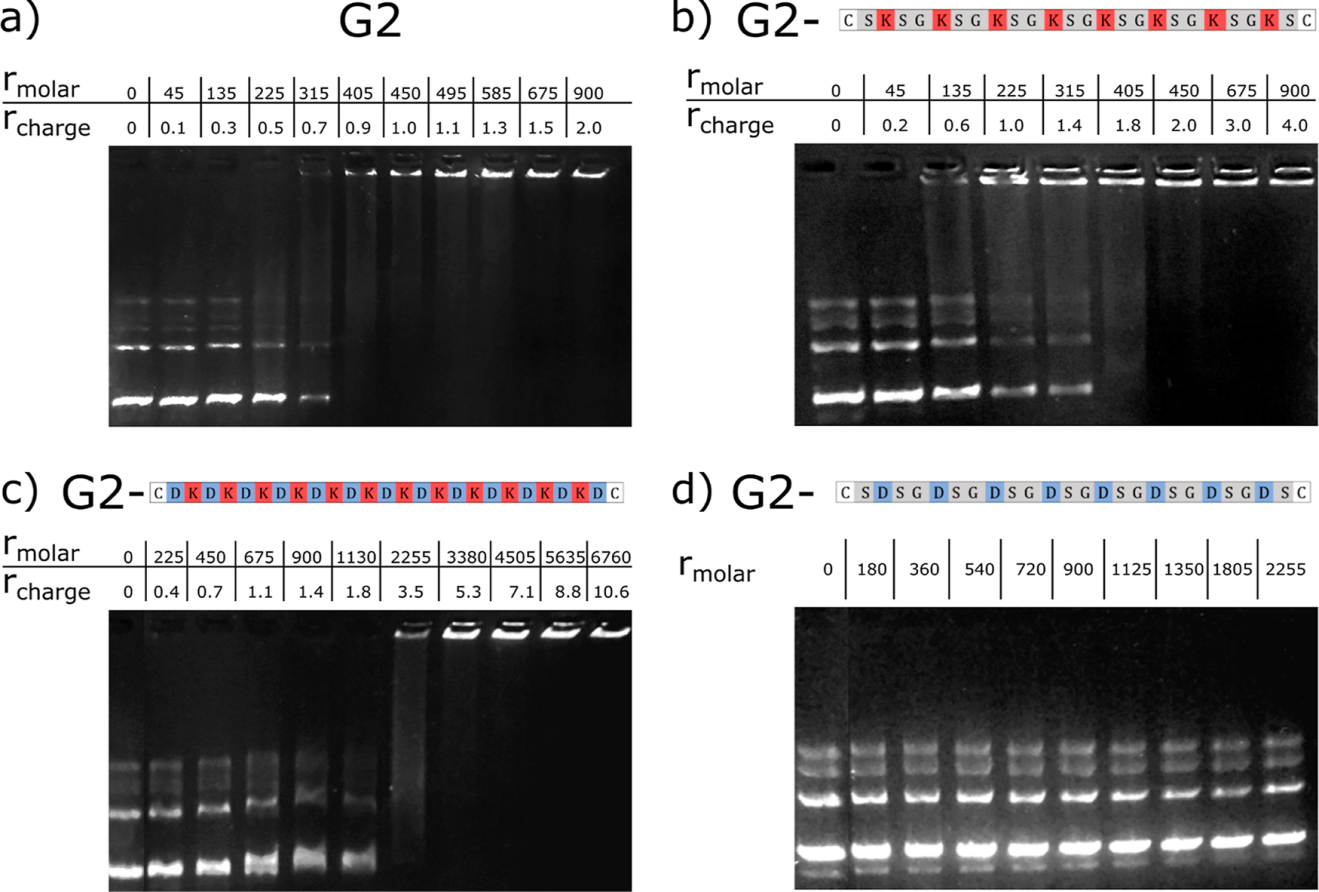
Gel electrophoresis images showing DNA mobility in the presence of (a) G2 dendrimers, (b) G2-(00*P*)_8_ conjugates, (c) G2-(*NP*)_12_*N* conjugates, and (d) G2-(00*N*)_8_ conjugates. The DNA concentration was 10 μg/mL, and the relative concentration of PAMAM dendrimers and conjugates is shown in *r*_molar_ and *r*_charge_.

Gel electrophoresis was used to probe DNA condensation mediated by G2 PAMAM dendrimers (Figure 3a). The first lane, containing only DNA, shows a strong band for the supercoiled DNA plasmid with the expected bp dimension (ladder not included in the image) and weaker bands corresponding to the relaxed plasmid and linear forms of the DNA. When the concentration of PAMAM is increased, a decrease in the intensity of the band attributed to the DNA and the appearance of a band in the wells are observed, indicating the coexistence of free and complexed/condensed DNA molecules. A *r*_charge_ of approximately 0.5 is enough to decrease the electrophoretic mobility of some DNA molecules, in good agreement with previous results showing complexation of DNA with G2 dendrimers at this mixing ratio.^42^ This suggests that we do not lose much G2 during the dialysis procedures. At *r*_charge_ = 0.9 almost all DNA is present in DNA-dendrimer complexes and for higher charge ratios, the DNA shows low electrophoretic mobility and remains in the wells. Such retardation of the DNA transport along the agarose gel as the concentration of G2 is increased can be due to charge neutralization of the DNA, increase in molecular weight of the DNA complexes due to G2 binding, and/or the formation of aggregates that are too large to travel through the pores of the gel.^43^

The electrophoresis experiments show the following trends regarding the impact of peptide conjugation on the interactions between DNA and G2. The steric effect of the G2 tails is first probed by considering tails with neutral a.a. (0_25_) or ampholytic tails with alternating negative and positive a.a. [(*NP*)_12_*N*]. While G2 induced DNA condensation at *r*_charge_ ≈ 0.9, G2-(*NP*)_12_*N* seems to hinder DNA condensation up to a charge ratio of *r*_charge_ ≈ 3.5 (compare Figure 3a with 3c). A similar result is obtained for the G2-0_25_ system (Figure S5).

Conjugating negatively charged peptide tails to G2 dendrimers seems to prevent the interaction of the dendrimers with the DNA, as the mobility of the DNA along the gel is not changed (see Figures 3d and S5b). This is not surprising since, as indicated in Table 1, the overall charge of the conjugates was *Z*_G2+2tail_ = −8.

While conjugation of uncharged or ampholytic peptides to G2 led to a higher *r*_charge_ needed to induce DNA complexation, when compared to G2, conjugates with (approximately) two positively charged tails reduce the required charge ratio back to *r*_charge_ ≈ 0.6 to retain some DNA molecules in the wells (see Figure 3b showing results for G2-(00*P*)_8_). This is similar to G2 dendrimers and lower than the corresponding peptides alone (see below). Tails with charge blocks, (*P*)_4_0_17_(*P*)_4_ and 0_8_*P*_8_0_8_, seem to be slightly less efficient at condensing DNA, requiring a charge ratio of *r*_charge_ ≈ 1 to retain the DNA in the wells (Figure S5d,e).

Both G2 and G2 conjugates with positively charged tails induce DNA condensation at *r*_charge_ ≈ 1; however, as the G2 conjugates have a larger valency, fewer molecules are required when compared to the G2.

In control experiments, the ability of the (nonconjugated) peptides to condense DNA was also probed. As expected, neutral or negatively charged peptides do not affect DNA conformation (Figure S6). On the other hand, positively charged peptides alone lead to DNA condensation, with the *r*_charge_ of peptides needed to achieve full DNA retardation depending mainly on their overall charge (Figure S7). This is not surprising, as in aqueous solutions, a multivalency of three is enough to induce some DNA condensation.^44−48^ System (0*P*)_12_0 (*Z*_tail_ = +12) is the more efficient peptide, showing a coexistence of free and complexed DNA molecules in the *r*_charge_ range 0.9–1.2. For the peptides possessing eight positive charges, a slightly larger peptide concentration is required to immobilize the DNA molecules in the wells, and only small differences are observed with the peptide charge distribution. While the (00*P*)_8_ system shows coexistence of free and complexed DNA molecules in the *r*_charge_ range 1.3–1.5, for systems (*P*)_4_0_17_(*P*)_4_ and 0_8_*P*_8_0_8_ the coexistence is most prominently seen between *r*_charge_ values of 1.1 and 1.5.

#### Dye Exclusion Assays

**Figure 4:**
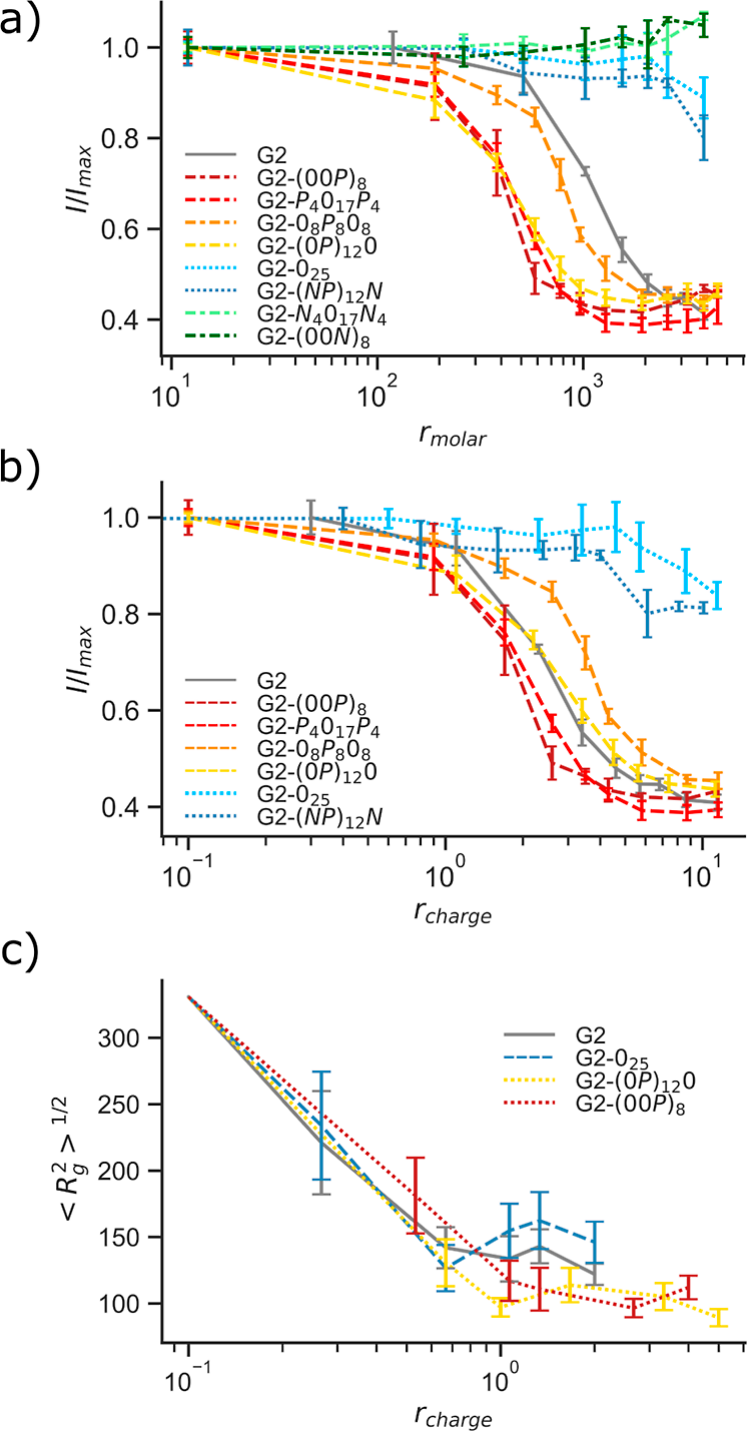
(a,b) Dye exclusion assays. Fluorescence intensity of GelStar is shown as a function of (a) *r*_molar_ and (b) *r*_charge_. Data are normalized to the fluorescence intensity of GelStar in samples containing only DNA (in the absence of G2 or G2 conjugates). Panel (c) shows the rms *R*_g_ of the model DNA as a function of *r*_charge_, evaluated using MC simulations. The error bars represent the standard deviation based on (a,b) triplicates and (c) at least six independent simulations. Lines between the data points are a guide to the eyes.

Dye exclusion assays were conducted using GelStar, which is a widely used technique to assess the availability of the DNA molecules to small molecules, based on differences in the fluorophore quantum efficiency of the dye in different environments. When GelStar binds to DNA in solution, its fluorescence emission intensity, quenched in an aqueous environment, increases. The association of condensing agents to DNA excludes the dye from the DNA molecules, leading to a decrease in the fluorescence intensity that can be easily monitored. Figure 4a,b shows the normalized fluorescence intensity of aqueous DNA-GelStar solutions at increasing concentrations of PAMAM and conjugated PAMAM dendrimers. Starting with the (nonconjugated) PAMAM dendrimers (gray curve), it can be seen that the intensity of the fluorescence signal emitted by GelStar decreases when the concentration of G2 PAMAM dendrimers is increased, indicating the exclusion of the dye from the DNA and DNA condensation. At *r*_molar_ = 1547 (*r*_charge_ = 3.4), the normalized fluorescence intensity is decreased to 0.55, and the lowest intensity is reached at *r*_molar_ ≈ 3000 (*r*_charge_ ≈ 5). It can be noted that the PAMAM concentrations required to achieve maximum dye exclusion are larger than those needed for full DNA retention in the gel electrophoresis wells. This could be due to the difference in DNA concentration used in the two experiments (2 *vs* 10 μg mL^–1^) and/or the differences between the measured quantities and experimental setups. While gel electrophoresis probes a more global macromolecular state, the exclusion of the dye can be a local effect and thus proceeds more gradually.

It is also interesting to compare these results with published work probing DNA condensation using G4 PAMAM dendrimers^14,49^ (with 64 charged end groups), where it was found that a *r*_charge_ ≈ 1 was sufficient for complete exclusion of the dye from the DNA. Lower concentrations of condensing agents with higher multivalency are usually required to induce similar levels of condensation, particularly in the dilute regime.^50−52^ Furthermore, the G2 PAMAM dendrimers are not able to exclude all dyes, indicating that the DNA is still accessible to small molecules.

Regarding the peptide-conjugated dendrimers, there is a clear distinction between the systems into three groups. As shown in Figure 4a, G2-0_25_ and G2-(*NP*)_12_*N* conjugates, with overall neutral (or close to neutral) peptide tails, are much less effective in preventing the binding of GelStar to DNA than PAMAM, with about 80% of the DNA being available for binding even at the highest studied *r*_charge_. While the gel electrophoresis shows retention in the wells for *r*_molar_ ≈ 3300 (*r*_charge_ = 6.4), about 80% of the DNA is still available for GelStar binding in the dye exclusion assay. This difference is discussed below.

Negatively charged tails prevent PAMAM conjugates from displacing GelStar dye altogether. As discussed above, these overall neutral or negatively charged conjugates do not strongly associate with DNA, despite the large inhomogeneity in their charge distribution. That is, the positively charged core is efficiently shielded in its capacity to interact with DNA by the negatively charged tails, independently of the distribution (block or alternating) of the negatively charged a.a.

All G2 conjugates with positively charged tails were able to prevent GelStar from binding to DNA at a low molar ratio. However, only two of the G2 conjugates [(00*P*)_8_ with alternating charges and *P*_4_0_17_*P*_4_ with charged blocks at each end] were slightly more efficient in condensing DNA than G2 alone, when considering the charge ratio, see Figure 4a. On the other hand, system G2-(0)_8_*P*_8_0_8_ appears to be less efficient in excluding GelStar (orange curves) than the other G2 conjugates with positive tails.

#### Dynamic Light Scattering

**Figure 5:**
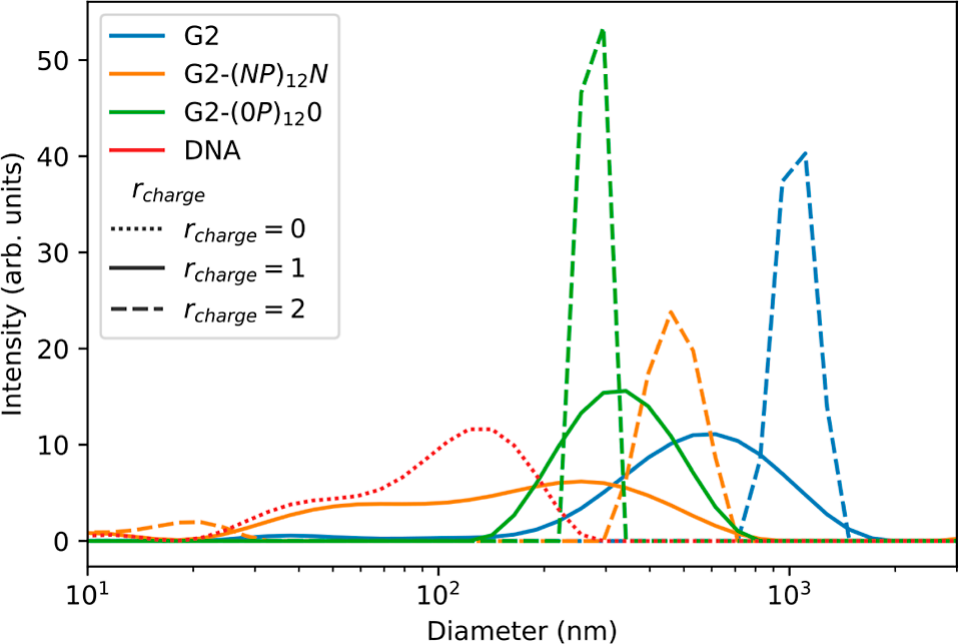
Size distribution of the hydrodynamic diameter of DNA in the absence and presence of G2 or G2 conjugates at *r*_charge_ = 1.0 and 2.0, as indicated.

DLS was used to evaluate the formation of polyplexes upon the addition of G2 and G2 conjugates. Figure 5 shows the size distributions of the DNA molecules and polyplexes evaluated using the software provided with the instrument. The size distribution for DNA is very broad and looks bimodal, which may appear unexpected, considering that we are working with DNA molecules with a fixed number of base pairs. Previously reported DLS measurements on DNA solutions have shown correlation functions exhibiting two or more relaxation modes, reflecting internal dynamics, in addition to the translational diffusion of DNA, for scattering angles above 57°.^53,54^

The addition of G2 to the DNA leads to an apparent increase in its hydrodynamic diameter and, more interestingly, to a decrease in the width of the size distribution and the disappearance of the second peak. This indicates a decrease in the internal dynamics of the DNA molecules, reflecting a more uniform complex.^49^ The apparent hydrodynamic radius of these complexes is very large, particularly for *r*_charge_ = 2.0, which suggests macromolecular aggregation. When zwitterionic peptide tails are attached to G2, the complexation of the DNA is hindered, as can be appreciated by the bimodal size distribution of the samples with *r*_charge_ = 1.0 (orange solid curve). At *r*_charge_ = 2.0, the distribution shows an apparent hydrodynamic diameter that is smaller than that of the DNA-G2 polyplexes, in addition to a small peak at around 20 nm, which is likely due to free G2 conjugates. The presence of charged peptide tails restores the condensation ability of the G2 dendrimers, in good agreement with the gel electrophoresis and dye exclusion assays. It is also seen that these polyplexes have a diameter smaller than that formed with G2 or G2-(*NP*)_12_*N*, with an apparent diameter of around 300 nm.

#### Monte Carlo Simulations

MC simulations have also been used to further assess DNA condensation by conjugated dendrimers, by probing the radius of gyration *R*_g_, defined as
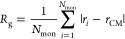
2where *r*_*i*_ and *r*_CM_ are the positions of monomer *i* and of the center of mass of the polymer, respectively.

**Figure 6:**
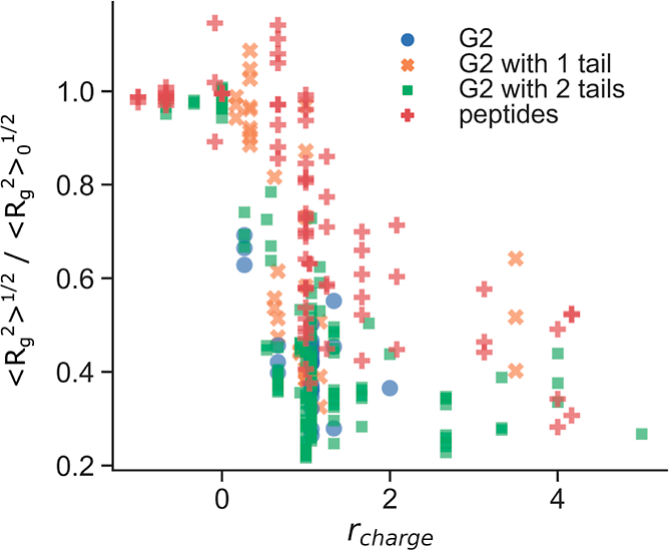
Scatterplot of the normalized rms of the radius of gyration of DNA as a function of the charge ratio, in the presence of G2 (blue circles), G2 with one and two conjugated peptides (orange crosses and green squares, respectively), and free peptide tails (red crosses).

The scatter plot in Figure 6 gathers the root-mean-square (rms) *R*_g_ of the model DNA in the presence of G2, peptides, and peptide-conjugated G2 with one or two tails of different compositions, as a function of *r*_charge_, defined in eq 1. The data are normalized by the rms *R*_g_ of the DNA alone .

As expected, overall negatively charged peptides and dendrimer conjugates (*r*_charge_ < 0) have a negligible effect on the DNA conformation. Some systems calculated at *r*_charge_ just above 0 show *R*_g, norm_ values above unity, indicating that the DNA molecule is more extended than the free DNA. This has been observed previously and has been attributed to the association of few condensing agents to the center of the chain, which extends the ends of the chains.^51^ It is also interesting to note that for 0.5 < *r*_charge_ < 1.25, there is a large dispersion of *R*_g, norm_ values, which is also found in the experiments around the extended-to-condensed (DNA) transition (*e.g.*, larger error bars in the dye exclusion assays and coexistence of seemingly free and condensed DNA molecules in gel electrophoresis). Independently of the used system, the transition from extended to more compact DNA conformations occurs at *r*_charge_ ≈ 1. The peptide tails alone (red crosses) seem to be less efficient in condensing the DNA than systems with dendrimers (blue circles), in good agreement with the gel electrophoresis experiments (see Figure S7). We recall that the overall charge of all of the studied peptide tails is lower than that of the dendrimers. There is also a larger spreading in the *R*_g, norm_ in systems where free peptides are present, compared to systems with conjugated dendrimers, for the same *r*_charge_, highlighting that the composition of the peptides (overall charge and charge distribution) has a larger impact when the condensing agents are less efficient. Furthermore, it can be observed that the conjugated dendrimers generally give rise to more compact DNA conformations, particularly those containing two tails, as can be seen by the concentration of green squares at low *R*_g, norm_ values (Figure 6).

The *R*_g, norm_ of DNA for selected systems at *r*_charge_ ≈ 1 is shown in Figure S8, which further highlights the effect of dendrimer conjugation on the DNA extension. It can be clearly seen that the peptides do not affect the DNA conformation to a large extent (red bars) and that the peptides with larger charge density are more efficient in condensing DNA. Regarding the peptides with an overall valence of *Z* = +8, collecting the positive charge in the block as opposed to a homogeneous distribution seems to slightly increase its condensing ability. These results are in good agreement with those from the gel electrophoresis experiments. G2 at *r*_charge_ ≈ 1 significantly reduces the extension of the DNA. The conjugation of 1 tail does not affect the DNA extension within the studied conditions but we recall that fewer vectors are used for the same *r*_charge_ (see snapshots in Figure S10a,b). The addition of a second peptide tail to the G2 slightly decreases the *R*_g, norm_ of the DNA (and again, fewer condensing agents are used), with the exception of the system with neutral tails (*O*_25_) where a small increase is observed instead.

Focusing on selected systems, Figure 4c highlights the decrease in the rms *R*_g_ of the model DNA as a function of G2 concentration, expressed as *r*_charge_. The conjugation of two positively charged tails to G2 results in a decrease in the number of dendrimers needed to induce a similar condensation degree as G2, but plotting the data as a function of *r*_charge_ (Figure 4c) reveals that the conjugated dendrimers are as efficient at condensing DNA as G2, at least to a *r*_charge_ ≈ 1, as also discussed above. In accordance with the gel electrophoresis results, *r*_charge_ ≈ 1 is enough to condense the DNA for almost all conjugated dendrimers. Interestingly, and unlike the results from the experimental work, adding two neutral tails to the G2 does not hinder the condensation of DNA induced by the dendrimer (blue *vs* gray curves in Figure 4), even if the rms *R*_g_ values appear to be a little larger than those of the other studied systems.

It is also interesting to look into the influence of G2 and DNA on the extension of the peptide tails. Figure S9 collects the rms *R*_g_ of the tails for the different systems.

Focusing on tails composed of neutral amino acids, it is seen that there are no significant differences in the extension of the peptides whether or not they are attached to G2 or are in the presence or absence of DNA. The same behavior is observed for the ampholytic peptides [(*NP*)_12_*N*], but the overall rms *R*_g_ is smaller than for 0_25_.

Regarding the negatively charged peptides, it is observed that attaching the peptides to the (oppositely charged) G2 significantly reduces their rms *R*_g_ (red *vs* blue), independently of the overall charge and charge distribution. The presence of DNA does not have an impact on the extension of the negatively charged tails. It is interesting to note that conjugates with one tail only are still overall positively charged and can associate with the DNA (Figure S10c). It can be seen that for system (0*N*)_12_0, the rms *R*_g_ of the tail when G2 is bound to one tail only is smaller than for dendrimers with two tails. This can be due to the electrostatic repulsion between the two chains and a looser wrapping of the tails around the dendrimer in the overall negatively charged conjugates. This is true for systems with and without DNA, as the rms *R*_g_ of the tails is not affected by the addition of the DNA.

When positively charged tails are considered, the opposite is observed; that is, attaching the tails to G2 leads to their extension, due to electrostatic repulsions. This can also be seen in representative snapshots of G2 conjugates in Figure S11. When DNA is added to the systems, a small decrease in the rms *R*_g_ is seen (blue *vs* green bars). Comparing systems with G2 dendrimers conjugated to one or two tails (orange *vs* green bars), we observe that the extension of the tail is independent of the number of tails and charge distribution for the systems with overall 8 charged a.a.; however, for system (0*P*)_12_0, it is seen that the extension of the tails significantly decreases when two tails are attached to the dendrimer, resulting in a smaller extension than the free tail. Taking into account how the tails are distributed along the DNA, and between the dendrimers (see below), we suggest that the reduction in size is a consequence of the repulsion between conjugates complexed with the DNA.

With the exception of the 0_8_*P*_8_0_8_ (central positive block), a small decrease of the rms *R*_g_ of the free tails is observed in the presence of DNA (red *vs* purple bars) due to the reduction of the intrachain electrostatic repulsions. Such variations are more obvious the longer the chain is,^52^ which can justify the lack of change observed for the 0_8_*P*_8_0_8_, where the charge is concentrated in a short segment.

### Structure of DNA-Conjugated Dendrimer Complexes

The structure of the formed polyplexes was evaluated by using MC simulations and AFM.

To evaluate the conformational changes of the model DNA induced by the conjugated G2, the asphericity of the DNA, an order parameter that reflects spatial symmetry, was calculated according to^55,56^
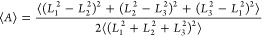
3where *L*_*i*_^2^, *i* = 1, 2, 3 are the eigenvalues in the tensor of the moment of inertia, which by definition are chosen as *L*_1_^2^ ≤ *L*_2_^2^ ≤ *L*_3_^2^. *A* takes the values [0,1] and can be used to assess the deviation from spherical symmetry, with *A* approaching 0 for spherical shapes while converging to 1 for rigid rods.

**Figure 7:**
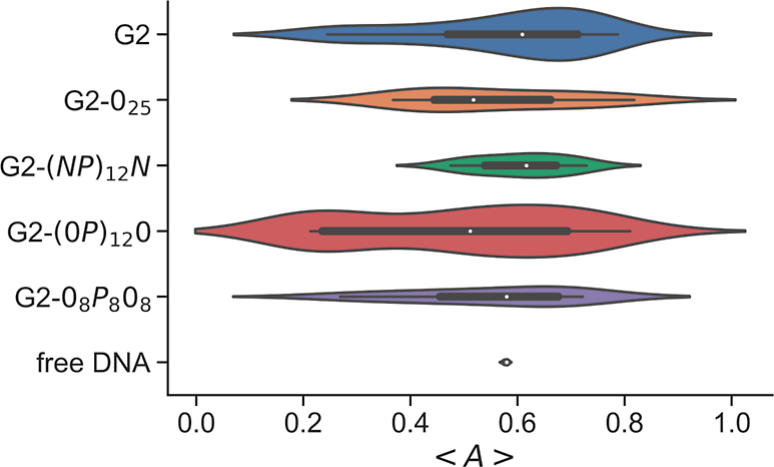
Asphericity of the model DNA for selected systems (indicated in the *y*-axis) with *r*_charge_ ≈ 1. The median asphericity is indicated by small white points, and the colored areas of the violin plot indicate the distribution of the mean asphericities calculated for each independent system.

It can be seen in Figure 7 that ⟨*A*⟩ = 0.578 ± 0.003 is obtained for the DNA. This value is larger than the value reported for self-avoiding chains, ⟨*A*⟩ = 0.534,^56^ which is to be expected from a relatively stiff polyelectrolyte chain.

**Figure 8:**
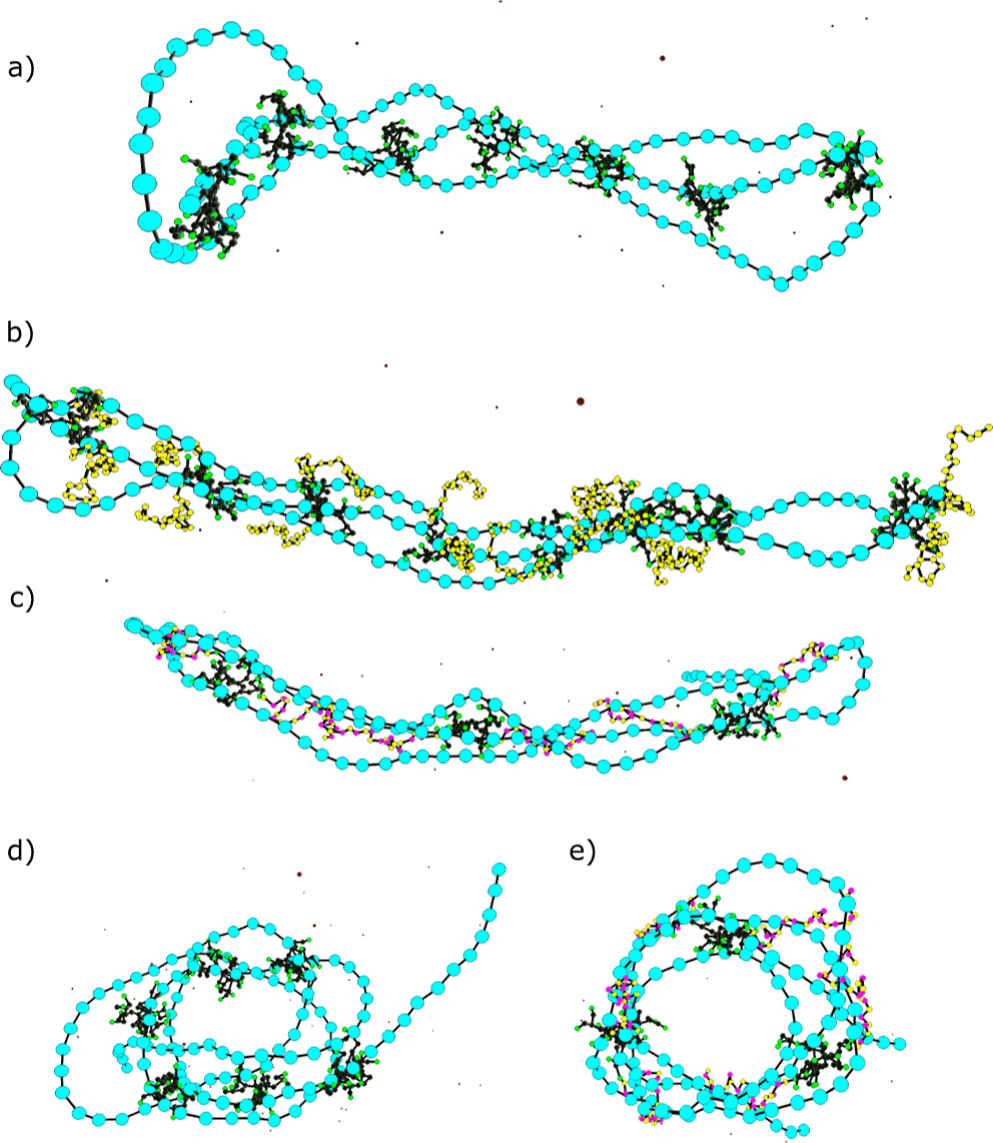
Snapshots of MC simulations showing the formation of polyplexes with *r*_charge_ ≈ 1 induced by (a) and (d) G2, (b) PAMAM-0_25_, and (c) and (e) G2-(0*P*)_12_0. DNA monomers are shown in turquoise, dendrimer monomers in green, neutral peptide chain beads in yellow, and positively charged peptide beads in pink.

The addition of G2 to the DNA results in significant changes to its asphericity, with the appearance of both toroidlike complexes, approaching the values expected from toroids (between 0.15 and 0.25),^57^ and rodlike structures, with values above 0.6.^57^ Representative snapshots of both types of structures can be found in Figure 8a,d. Even if the sampling of these structures was not large (20 independent runs), rodlike structures were more commonly found, as indicated by the width of the density plot around 0.7 (blue, Figure 7). These results are in good agreement with the cryo-TEM observation that low-generation PAMAM dendrimers (G1 and G2) induce the formation of rods and toroids.^13^ Adding neutral tails to G2 leads to structures similar to those of G2, as shown by the asphericity (Figure 7) and visual inspections of the snapshots (Figure 8b). There seems to be a lower probability of forming toroidal structures, particularly for the G2 conjugated with ampholytic peptide tails, however, a significant increase in the sampling would be necessary to draw a more definitive conclusion.

Systems with G2-(0*P*)_12_0 conjugates show a bimodal distribution of the asphericity, with maxima around 0.24 and 0.72, indicating the presence of both toroid- and rodlike conformations (Figure 8c,e). G2-0_8_*P*_8_0_8_ conjugates, on the other hand, do not show the same tendency to form toroids, which again could be due to a smaller sampling (6 versus 20 independent runs).

**Figure 9:**
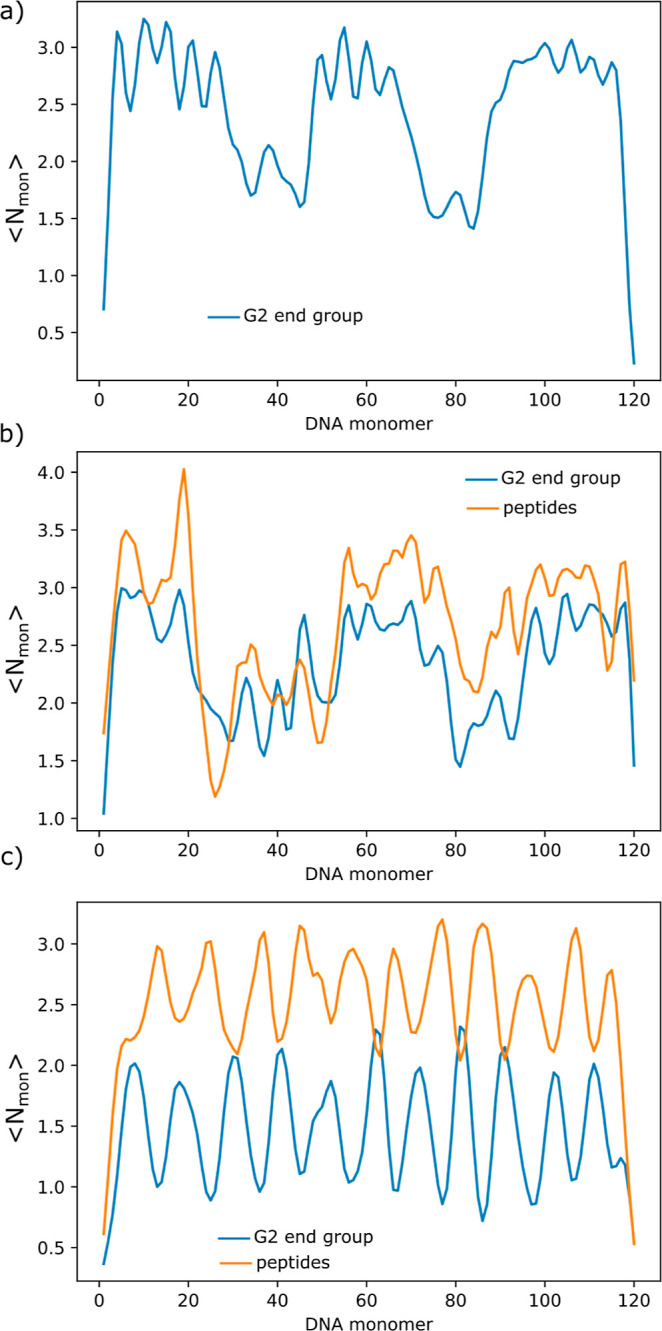
Average number of G2 end groups (blue curves) and peptides (when present, orange curves) in the vicinity of each DNA monomer versus the rank of DNA monomer for systems with (a) G2, (b) G2-0_25_, and (c) G2-(0*P*)_12_0, indicating the preferential position of the vector components along the DNA. All systems have *r*_charge_ ≈ 1 and the conjugates have two tails.

It is possible to study the distribution of the dendrimers along the DNA chain by assessing the average number of G2 end groups found in the vicinity (center-to-center separation of 20 Å) of each DNA monomer (Figure 9). For clarity, the presented plots correspond to individual runs and not to an average of independent runs. The main observations are not dependent on the initial conditions of the simulations but considering the average would even out some of the details. It can be seen that the G2 dendrimers are distributed with some regularity along the DNA chain, showing some repulsion between them and avoiding the ends of the chains. This has also been observed upon the association of macroions and short polycations onto oppositely charged polyelectrolytes.^51,58^ The areas around chain segments 40 and 80 that show a low probability of finding G2 end groups correspond to the turns of the rodlike structure, seen in Figure 8a. In configurations showing toroidal structures, the G2 are more uniformly distributed along the DNA. Figure 9b gathers the average number of both G2 end groups and monomer tails along the model DNA, for dendrimer conjugates with neutral tails (G2-0_25_). This particular system also corresponds to a rodlike conformation, and the average number of G2 end groups in the vicinity of the DNA is similar to that obtained for G2. However, the dendrimers seem to be more uniformly distributed along the DNA chain, potentially due to steric repulsions between the neutral tails of the associated dendrimers. It can also be seen that the contact profiles of the dendrimer end groups and tail groups are very similar. The situation is different when two positively charged tails are considered (Figure 9c). In this case, the ⟨*N*_mon_⟩ for the end groups is lower than for G2 and G2-0_25_ systems and that of the positive a.a. in the tails is higher compared to the end groups. Furthermore, it is clearly seen that the peptide tails occupy the space along the DNA left available by the G2. The decrease in the ⟨*N*_mon_⟩ of the end groups for system G2-(0*P*)_12_0 might be due to the competition for DNA binding and electrostatic repulsion from the positive tails. The distribution of the positively charged particles in the tail allows for a more efficient contact of these particles with DNA when compared to the distribution of the end groups in G2, which explains the larger ⟨*N*_mon_⟩ for the tails compared to the end groups of the G2.

**Figure 10:**
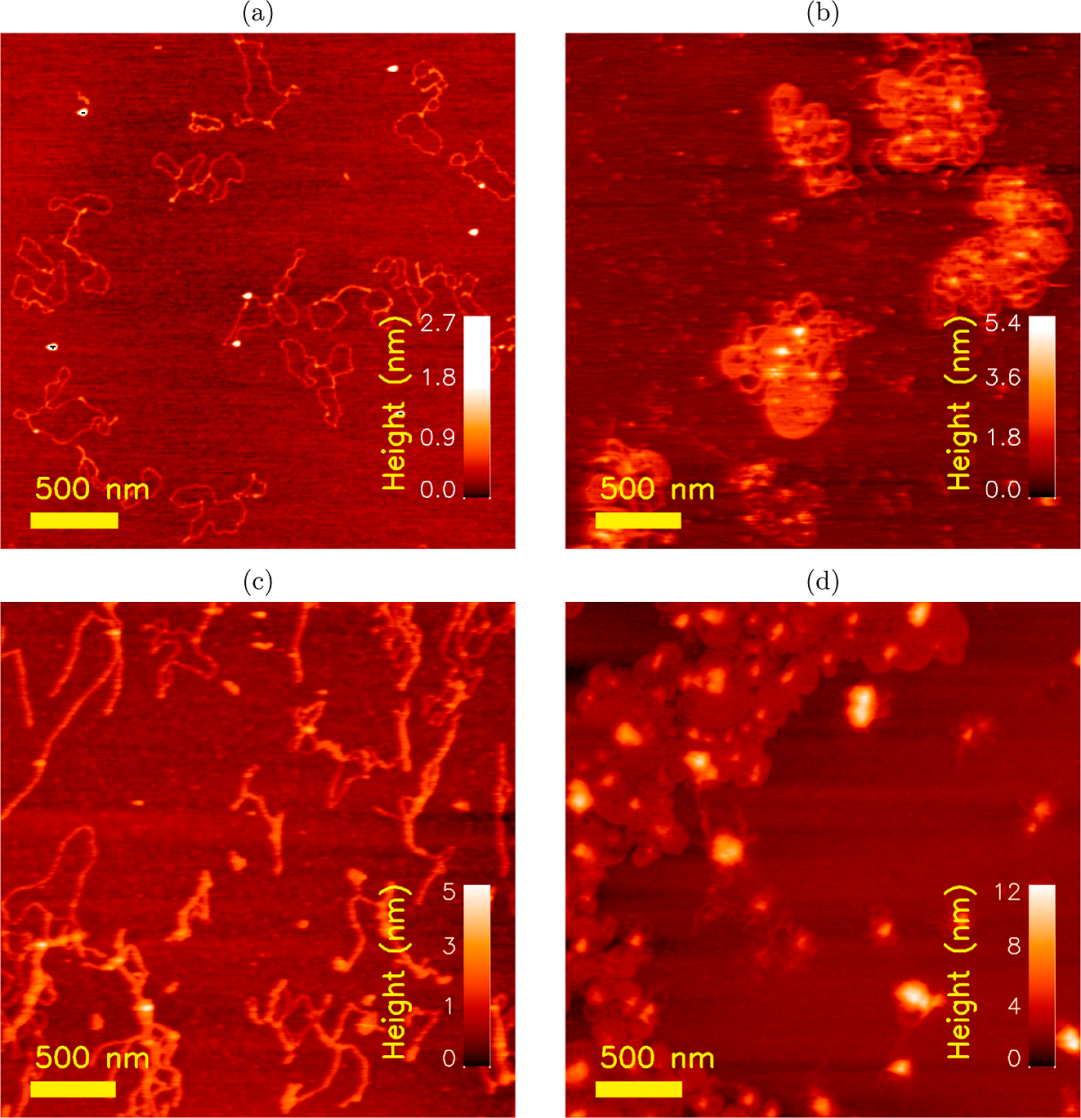
Tapping mode AFM height topographs of (a) plasmid DNA, (b) plasmid complexed with G2, (c) plasmid complexed with G2-(*NP*)_12_*N* peptide conjugates, and (d) plasmid complexed with G2-(0*P*)_12_0 peptide conjugates. The polyplexes were prepared at *r*_charge_ = 2.

AFM was used to assess the impact of the peptide tails on the structure of the polyplexes. Based on the results reported above, selected systems were imaged at *r*_charge_ = 2, where the majority of the DNA molecules are expected to participate in complex formation. AFM topographs reveal a plasmid that is adopting various extents of supercoiling (Figure 10a), similar to AFM images previously reported by Reitan *et al.*(59)

The DNA complexed with the G2 at *r*_charge_ = 2 (Figure 10b) shows more tightly nested structures where there are identifiable condensed regions surrounded by apparently uncomplexed parts of the DNA. The resulting overall appearance as a miniature skein of yarn is similar to structures previously reported for DNA complexation with other polyamines.^60−63^ The observed polyplex structures are not entirely consistent with the MC results shown above or the cryo-TEM visualization of similar systems (see Figure 6i,j in ref (13)). One of the primary differences between these is the DNA architecture (plasmid in the AFM versus linear in the MC simulations and cryo-TEM). In addition, and focusing on the experimental results, the sample preparations are necessarily different with a drying step in one and the vitrification in the other, the *r*_charge_ of the observed samples also differed (2.0 *vs* 0.5, for AFM and cryo-TEM, respectively), as well as the salt conditions. The AFM was used in salt-free samples to avoid the visualization of salt crystals upon drying. All these facts may have contributed to the observed differences, but it should also be stated that due to the difficulty in assessing the internal structure of the complexes from the AFM topographs, the presence of toroidal and rodlike structures within the larger aggregates cannot be completely ruled out. The AFM topographs of the DNA mixed with the G2-(*NP*)_12_*N* ampholytic peptide (Figure 10c) reveal polyplexes not as condensed as observed for G2 alone, but there is also more apparent cluster formation as compared to the uncomplexed DNA (note the differences in the height scale). This finding indicates that the conjugation of the ampholytic peptides to the G2 reduces the complexation ability with DNA, although the net charge balance is maintained. Increasing the net positive charge of the conjugated peptide to the G2 (while keeping *r*_charge_ = 2) resulted in restoring the complexation capacity of G2 with the DNA as indicated by the AFM topographs. Additionally, there appears to be a stronger interaction between the DNA and G2-(0*P*)_12_0 (Figure 10d) as compared to G2, as indicated by the denser structures observed in the AFM topographs.

Taking all results together, we can summarize that (i) attaching neutral peptides to G2 dendrimers hinders to some extent the formation of polyplexes, (ii) negatively charged tails prevent the interaction between G2 and DNA, but (iii) if (some of) the a.a. are positively charged, the interaction between the macromolecules and the formation of polyplexes is restored. While the results from all studied techniques are consistent in points (ii) and (iii), some discrepancy was found between the experimental and modeling results regarding point (i). In short, gel electrophoresis showed that large *r*_charge_ values of the conjugates with neutral tails are required to prevent diffusion of the DNA in the gels when compared to G2 but did not prevent it completely within the studied concentration range. Dye exclusion assays suggest that about 80% of the DNA is accessible to the dye at *r*_charge_ values of up to 10. On the other hand, the neutral tails of the dendrimers did not prevent their association with the DNA, according to the simulations, and the DNA was shown to have a more condensed form than free DNA, using DLS, simulations, and AFM. Taking these results into account, it is suggested that the neutral tails do not directly prevent association between the G2 and the DNA but since they extend from the polyplex, the association of several DNA molecules and formation of polyplexes involving multiple DNA chains is hindered due to steric effects. We further suggest that such polyplexes involving few DNA molecules are more susceptible to binding from small molecules, justifying the results from the dye exclusion assays.

## Conclusions

PAMAM dendrimers are promising molecules for nucleic acid delivery. Inspired by the role of disordered peptide tails in DNA–protein interactions, we conjugated peptide tails to dendrimers with the purpose of tuning the interaction between PAMAM and the nucleic acids.

It was found that conjugating neutral peptides to G2 PAMAM dendrimers leads to the formation of polyplexes composed of few DNA molecules, which was attributed to the steric effects resulting from the extension of the neutral tails from the DNA when the G2 associated with it. This makes DNA more accessible to small molecules. The presence of two negatively charged tails renders conjugated G2 neutral, which prevents the interactions between G2 and DNA. On the other hand, increasing the net positive charge of the conjugated peptides increases the overall charge of the conjugates and restores the interactions of G2 with DNA and formation of polyplexes involving multiple DNA molecules. While DNA complexation is obtained for a similar net charge balance for G2 and G2 conjugated with positive tails, fewer of the latter are required to achieve a comparable condensation degree. This implies that fewer G2 dendrimers are required to achieve DNA condensation when conjugated to peptide tails. Furthermore, the positive tails tend to occupy the space along the DNA left free by the G2, which also reduces the average number of G2 end groups in close proximity to DNA. Nevertheless, about 40% of the DNA remains accessible to small molecules, which can be advantageous for applications such as nuclei acid delivery.

## Supporting Information Available

Plasmid map of DNA used in experiments; details on the conjugation of peptides to PAMAM and characterization of PAMAM-peptide conjugates; supporting figures of gel electrophoresis results; details on the model parameters and interaction potential of MC simulations; supporting figures showing the radius of gyration of DNA and peptides for several systems; and more snapshots of the simulations (PDF)
Gene bank sequence of the plasmid DNA used in the dye exclusion assays and gel electrophoresis (TXT)
