# Supplementary material for: DNA Condensation by Peptide-Conjugated PAMAM Dendrimers. Influence of Peptide Charge
Source: ACS Omega. 2023 Nov 15;8(47):44624–36. doi: 10.1021/acsomega.3c05140 (PMC10688094; doi:10.1021/acsomega.3c05140)
Supplement: Supplementary file 1 — ao3c05140_si_001.pdf [file ao3c05140_si_001.pdf]

Supplementary Information

for

DNA condensation by peptide-conjugated PAMAM dendrimers. Influence of peptide charge

Corinna Dannert,† Ingrid Mardal,† Rahmi Lale,‡ Bjørn Torger Stokke,† and Rita S. Dias*,†

†Biophysics and Medical Technology, Department of Physics, NTNU - Norwegian University of Science and Technology, Trondheim, N-7491, Norway.

‡Department of Biotechnology and Food Science, NTNU - Norwegian University of Science and Technology, Trondheim, N-7491, Norway.

E-mail: rita.dias@ntnu.no

DNA plasmid sequence

The plasmid used in both dye exlcusion and gel electrophoresis experiments was 3605bp long, see fig. S1. The sequence was created in Benchling.

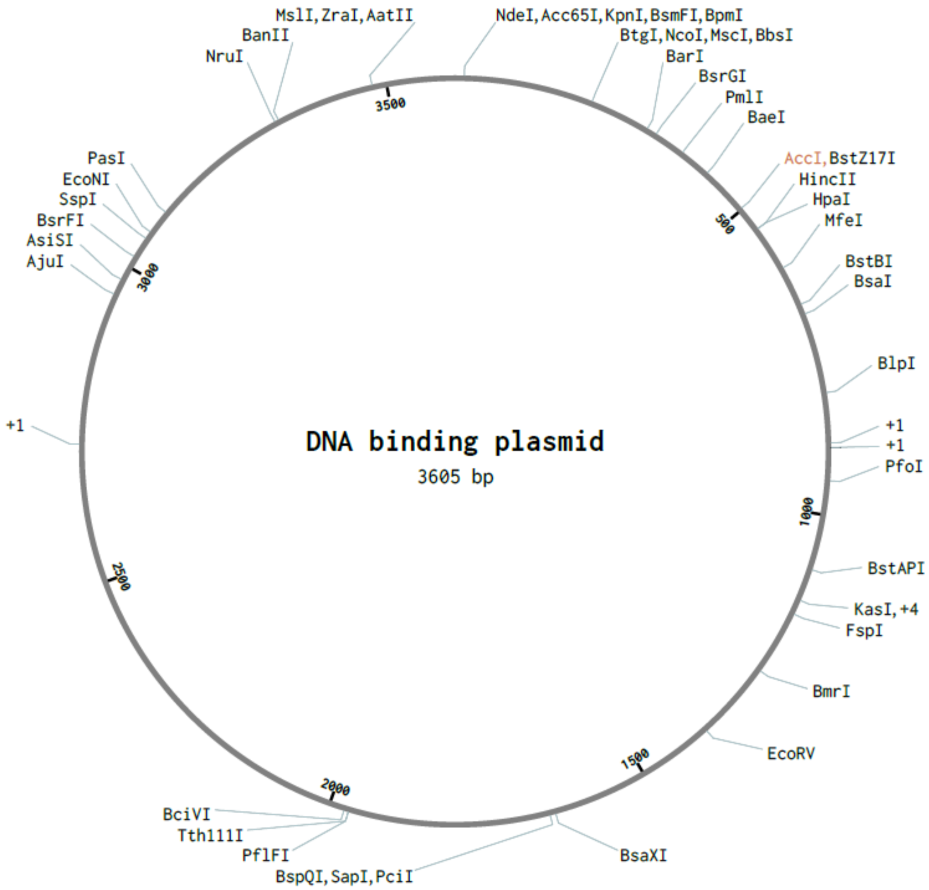

Figure S1: Scheme of plasmid used in dye exclusion and gel electrophoresis experiments.

Conjugation of PAMAM G2 and peptides

The protocols we followed for conjugating the peptides to dendrimers used G5 PAMAM dendrimers. Since G2 was used in this work, tests were conducted to find the ideal molar ratio between G2 and the SPDP linker to give about two tails per dendrimer. For this purpose, SPDP:G2 molar ratios of 3:1, 4:1, 5:1 and 6:1 were investigated. To quantify the average number of, firstly,

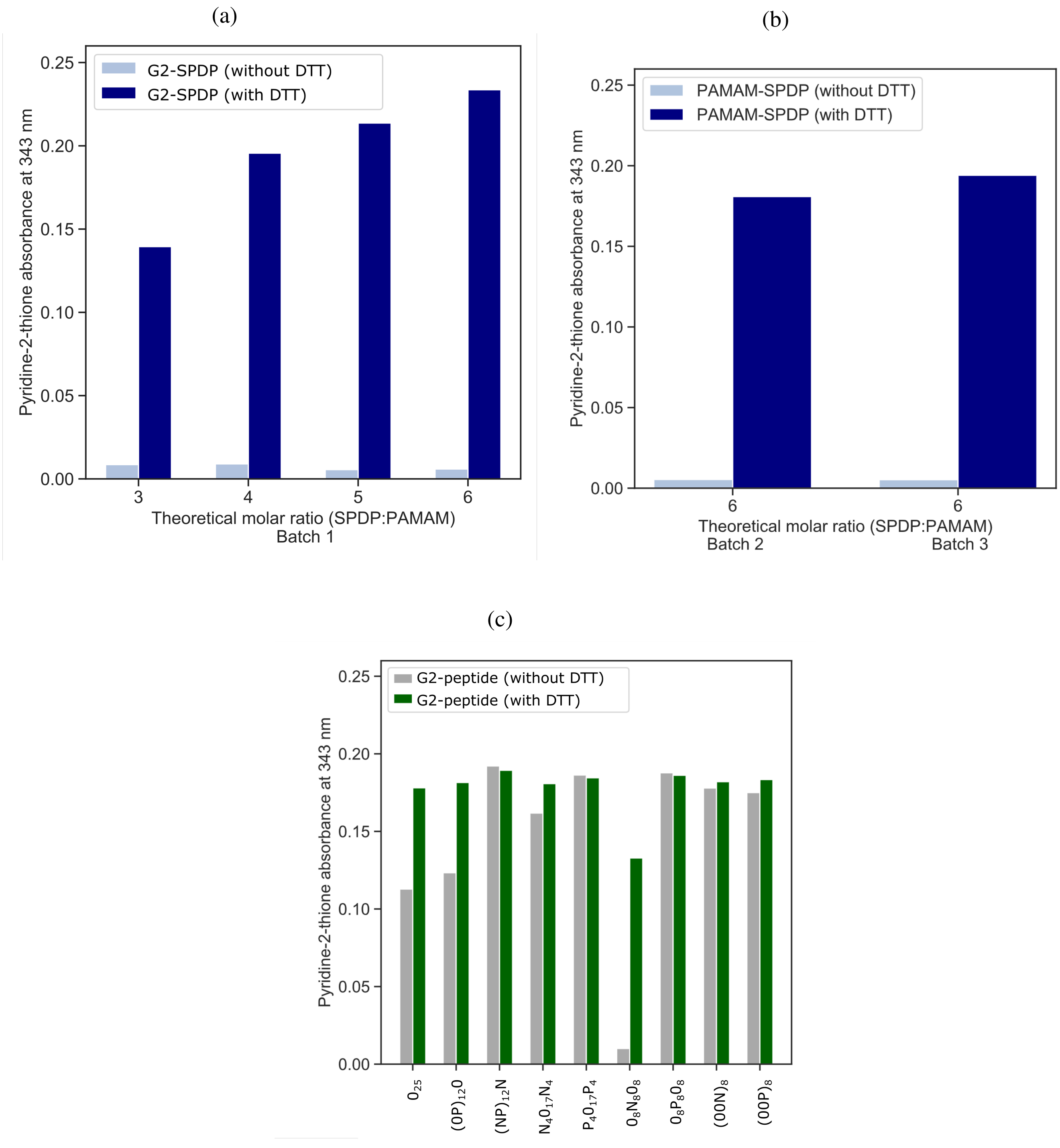

Figure S2: Pyridine-2-thione UV absorbance at 343 nm a) and b) after conjugating G2 PAMAM dendrimers with Sulfo-LC-SPDP linker before and after addition of DTT, and c) after conjugating G2-SPDP with peptides before and after addition of DTT.

SPDP linkers and, secondly, peptides conjugated to G2 dendrimers, DTT assays were performed, as shown in Fig. 1 and explained in the main text. As shown in fig. S2a, the absorbance upon addition of DTT increases for increasing SPDP:G2 molar ratios, which indicates that a larger number of SPDP covalently binds to G2 with increasing concentration of SPDP. The average number of conjugated SPDP per G2 increased from 1.5 to 2.6 when the molar ratio increased from 3 to 6.

The first batch was performed using a 800 µL solution of G2. Upscaling the process to a volume range of 6 mL to 10 mL and improving the dialysis conditions (increase in buffer volume and dialysis time) for SPDP:G2 molar ratios of 6:1, resulted in batches with similar absorbance of pyridine-2-thione at 343 nm, and thus similar average number of SPDP linkers per G2 of 2.3 (fig. S2b).

To evaluate the efficiency of peptide attachment to G2-SPDP conjugates, the number of conjugated peptides was measured using a DTT assay. Here the pyridine-2-thione released by the binding of peptides to the linker was assessed after the addition of peptide to the G2-SPDP conjugates (reaction C in Fig. 1, grey bars in fig. S2c). Then the absorbance of pyridine-2-thione was again measured after addition of DTT, which also included the released pyridine-2-thione from nonreacted SPDP groups (green bars in fig. S2c). Thus, an increase in absorbance after the addition of DTT indicates non-occupied SPDP linkers that have not reacted with a peptide.

Systems with the negatively charged peptide $0_8N_80_8$ showed very little absorbance before addition of DTT, indicating a very low amount of bound peptides, and was thus excluded from further experiments. Peptides 0_{25} and $(0P)_{12}0$ show a significant increase in absorbance after the addition of DTT, suggesting the existence of non-occupied SPDP groups.

The efficiency of the conjugation was also evaluated using ^{1}H NMR spectroscopy.

Due to the abundance of protons in both the G2, SPDP and peptide tails, it was difficult to definitively assign each peak in the NMR spectra to specific hydrogen atoms. However, it was possible to identify differences in the spectra of G2, G2-SPDP and G2 with peptide tails. An overview of the spectra of all measured samples (G2, SPDP, G2-SPDP, $(00P)_8$ and G2-$(00P)_8$ can be found fig. S3a.

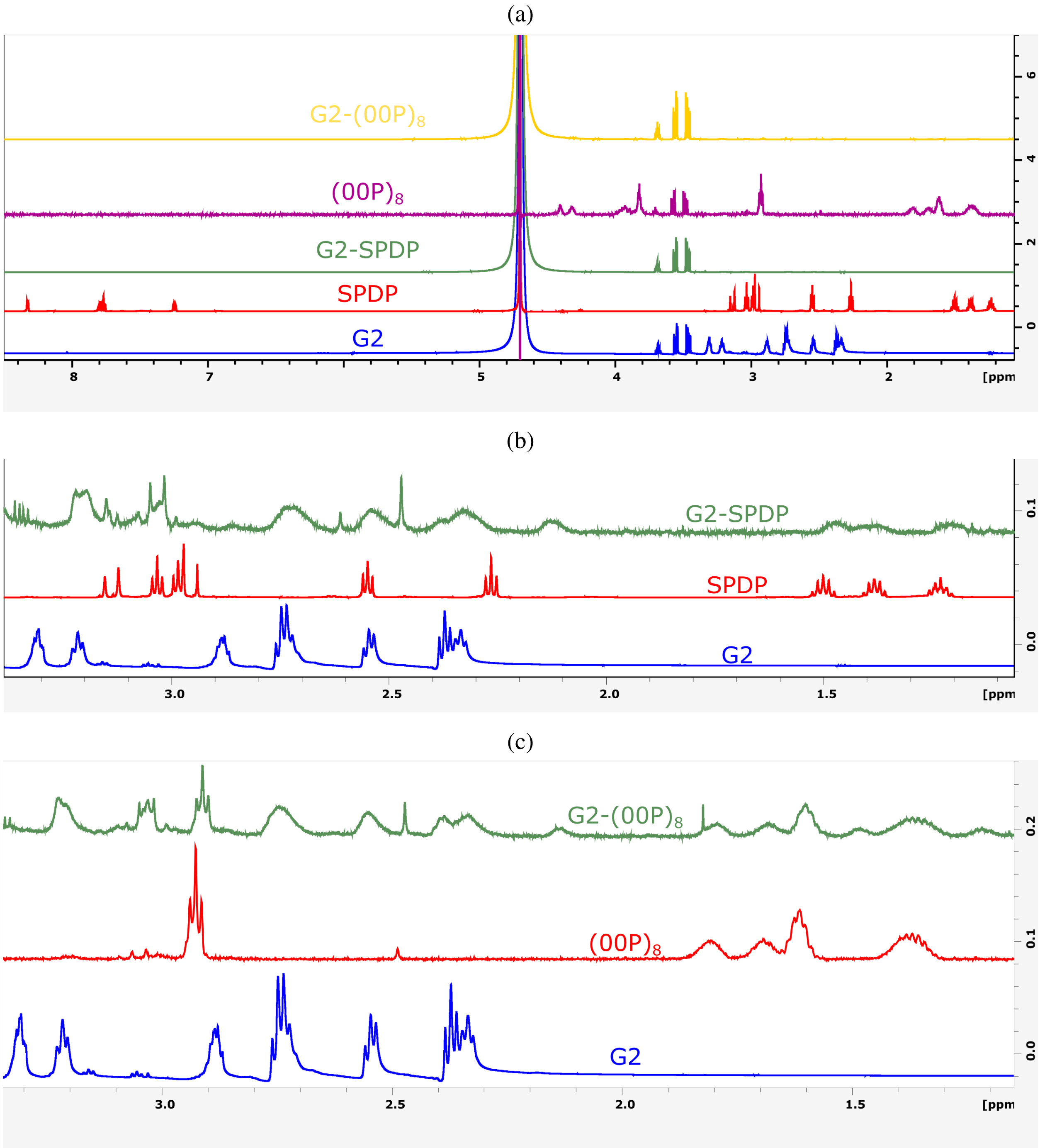

Figure S3: ^{1}H NMR spectra of a) G2, SPDP, G2-SPDP, $(00P)_8$ peptide and G2-$(00P)_8$, b) G2, SPPD, G2-SPDP and c) G2, $(00P)_8$ peptide and G2-$(00P)_8$.

Firstly, comparing the spectra of G2, SPDP, and G2-SPDP (fig. S3a) one can see that the multiplet peaks between $\delta = 7.2 - 8.3$ arising from the SPDP disappear in the spectrum of G2-SPDP. This indicates the successful cleaving of the succinimide ring of SPDP (see reaction A in

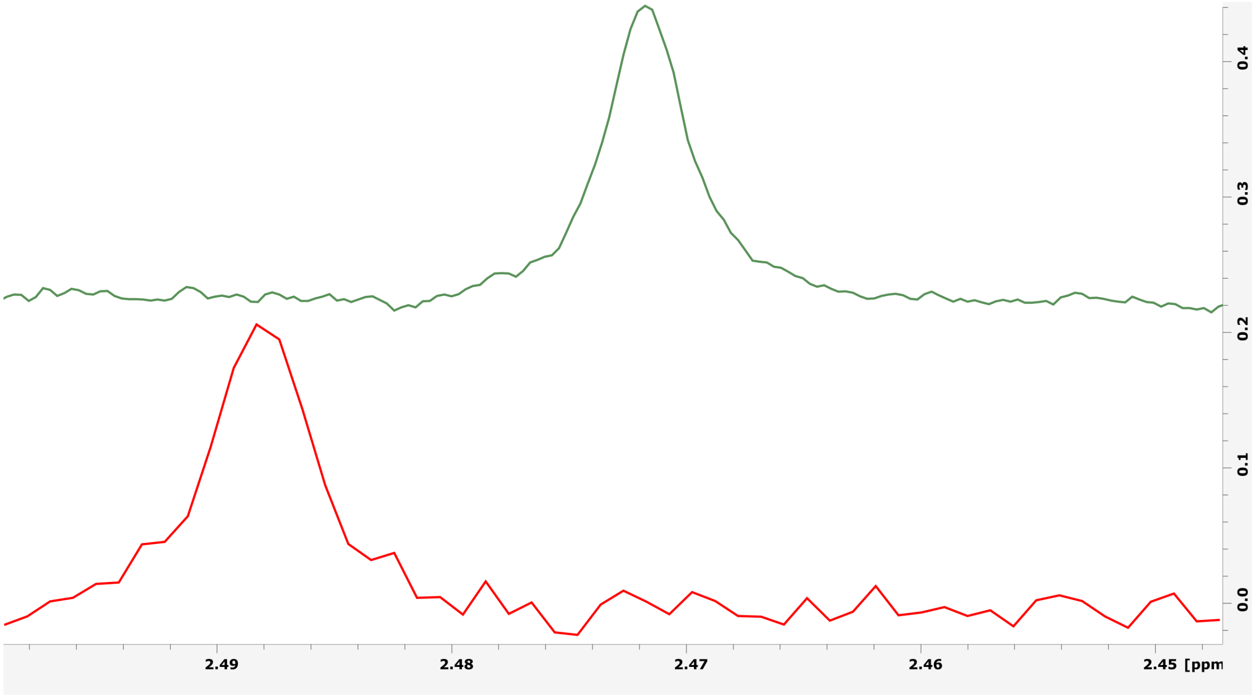

Figure S4: ^{1}H NMR spectra of $(00P)_8$ peptide and G2-$(00P)_8$ focused on a shift $\delta \approx 2.45 - 2.5$

Fig. 1). Furthermore, three broad peaks at $\delta = 1.21, 1.36, 1.48$ are identified in the spectrum of G2-SPDP, which are not present in the spectrum of G2 alone but stem from the SPDP (fig. S3b)).

Secondly, the spectrum of G2-$(00P)_8$) shows peaks from both G2 and the peptide $(00P)_8$ (fig. S3 c)). Based on the areas of the peaks in spectrum of $(00P)_8$, we determined that the peak at $\delta = 2.49$ belongs to the hydrogens in close proximity to the thiol group of the two cysteine residues in the peptide. This peak experiences a small shift to $\delta = 2.47$ in the spectrum of G2-$(00P)_8$ (fig. S4), indicating the successful formation of disulfide briges, and thus the conjugation to the SPDP bound to G2.

Gel Electrophoresis

fig. S5 shows the gel electrophoresis of DNA complexed with G2-conjugates. G2-0_{25} is much less efficient in retaining the DNA in the wells compared to G2 alone (fig. S5a) while G2-$N_4 0_{17} N_4$ does not retain the DNA in the wells even at high molar ratios (fig. S5b). Conjugates with positively charged tails (fig. S5c-e) all show similar behaviour to G2-$(00P)_8$, which was discussed in the main

text. Gel electrophoresis of DNA with neutral or negatively charged peptides is shown in fig. S6. Even at high concentrations, no DNA is retained in the wells, indicating a lack of binding of the peptides to the DNA. Positively charged peptides, on the other hand, do impact the mobility of the DNA through the gel, see fig. S7. Interestingly, peptides $(00P)_8$ (fig. S7a) and $0_8P_80_8$ (fig. S7d)) both show a significant retention of the DNA in the wells starting at r_{charge}=1.5, while $P_40_{17}P_4$, with the same peptide charge, shows retention already at r_{charge}=1.1. $(0P)_{12}0$ (fig. S7c) shows DNA retention in the wells already at a r_{charge}=0.9, similar to G2 dendrimers, due to the larger charge density of the peptides.

Monte Carlo Simulations

Model parameters and interaction potentials

The total potential energy of the system was calculated as the sum of bonded and non-bonded interactions $U = U_{\text{nonbond}} + U_{\text{bond}} + U_{\text{ang}}$.

The non-bonded potential U_{nonbond}, given as

$$U_{\text{nonbond}} = \sum_{i,j} U_{i,j}(r_{i,j}), \tag{S1}$$

is divided into a hard-sphere contribution and a screened Coulomb potential energy contribution, according to

$$U_{i,j}(r_{i,j}) = \begin{cases} \infty, & r_{i,j} < R_i + R_j \\ \frac{z_i z_j e^2}{4\pi\varepsilon_0\varepsilon_e} \frac{\exp(-r_{i,j}/l_{\text{sc}})}{r_{i,j}}, & r_{i,j} \geq R_i + R_j \end{cases} \tag{S2}$$

where z_i is the charge of a particle i, $r_{i,j}$ is the separation between particles i, j, R_i is the radius of particle i, ε_0 and ε_r are vacuum and relative permittivity, respectively, and l_{sc} is the Debye screening length. $\varepsilon_r = 78.4$ and $l_{\text{sc}} = 6.802\,\text{nm}$, describing an aqueous solution at 298 K with a (monovalent) salt concentration of 2 mM, were used throughout the work.

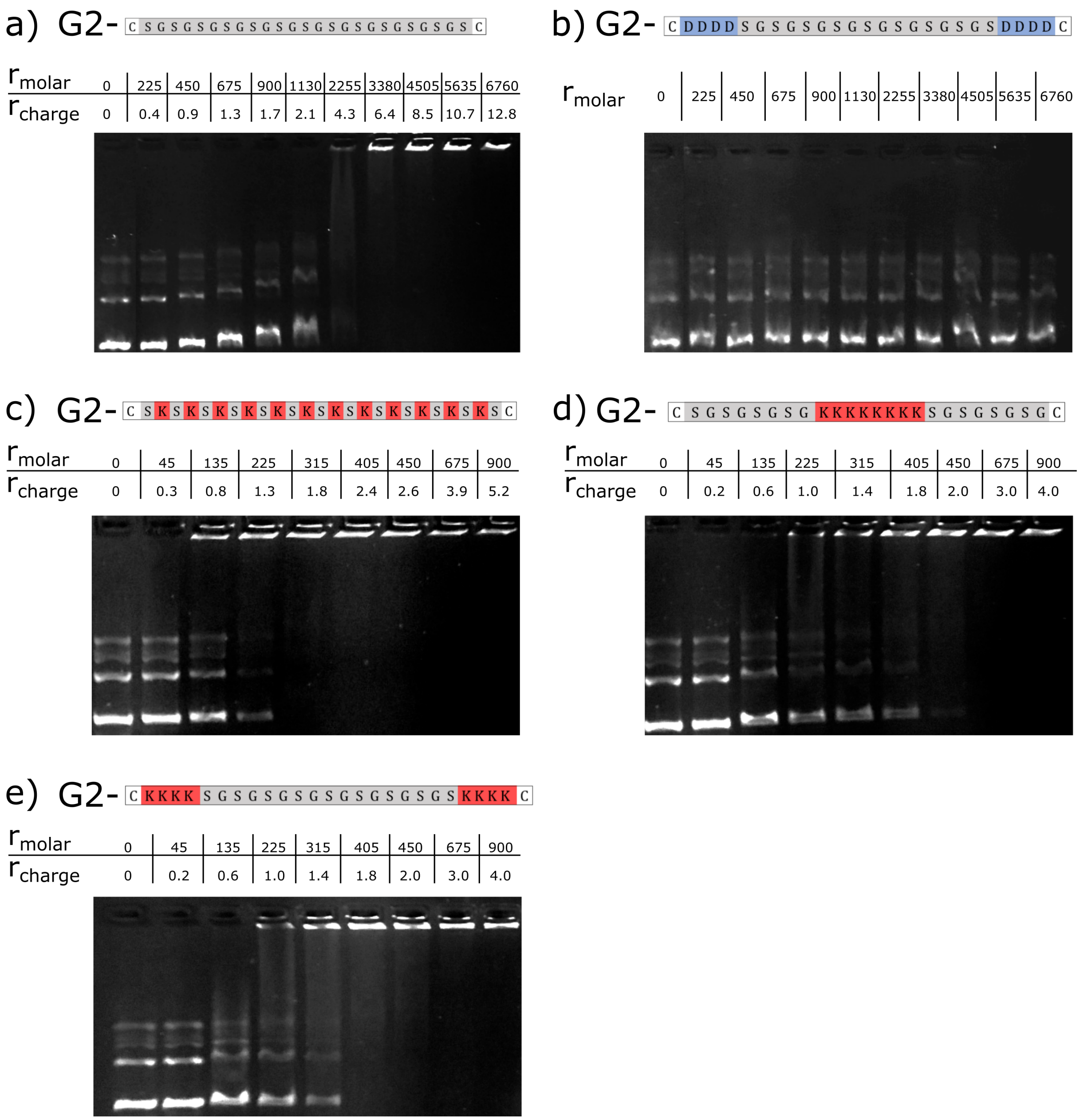

Figure S5: Gel electrophoresis of 10 µg/mL plasmid DNA with increasing concentrations of G2-conjugates. a) G2-0_{25}, b) G2-$N_4 0_{17} N_4$, c) G2-$(0P)_{12}0$, d) G2-$0_8 P_8 0_8$ and e) G2-$P_4 0_{17} P_4$.

The bond potential energy is given by

$$U_{\text{bond}} = \frac{k_{\text{bond}}}{2} \sum_{i}^{N_{\text{mon}}} \left(r_{i,i+1} - r_0\right)^2, \tag{S3}$$

where, $r_{i,i+1}$ is the distance between two connected monomers (bond length) with the equilibrium

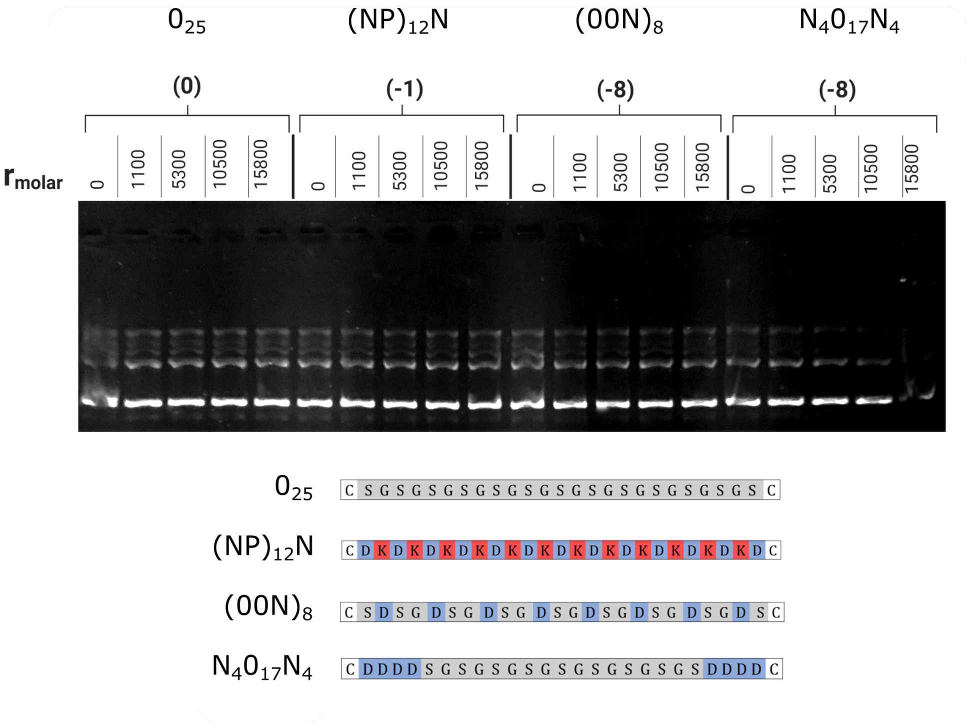

Figure S6: Gel electrophoresis of 10 µg/mL plasmid DNA with increasing concentrations of neutral (0_{25} and $(NP)_{12}N$) and negatively charged peptides, as indicated.

separation r_0 and the force constant k_{bond}. In this work, $r_0 = 5\,\text{Å}$ and $k_{\text{bond}} = 2.4\,\text{N/m}$ were used for the dendrimers and peptides, and $r_0 = 15\,\text{Å}$ and $k_{\text{bond}} = 10\,\text{N/m}$ for the DNA chain.

The angular potential energy, describing the intrinsic stiffness of the chains, is given by

$$U_{\text{ang}} = \frac{k_{\text{ang}}}{2} \sum_{i}^{N_{\text{mon}}-2} (\alpha_i - \alpha_0)^2, \tag{S4}$$

where, α_i is the angle formed by the vectors $\mathbf{r}_{i+1} - \mathbf{r}_i$ and $\mathbf{r}_{i-1} - \mathbf{r}_i$ with the equilibrium angle $\alpha_0 = 180°$. The angular constant $k_{ang} = 0.01\,\text{J/deg}^2$ for both the hierarchical structures (dendrimers and conjugated peptides) and the DNA chain.

Since the dimensions of toroids do not change significantly with the number of DNA molecules involved in the complexes, this work was conducted using a single chain system within a spherical simulation. This set-up avoids using periodic boundary conditions and the concomitant computa-

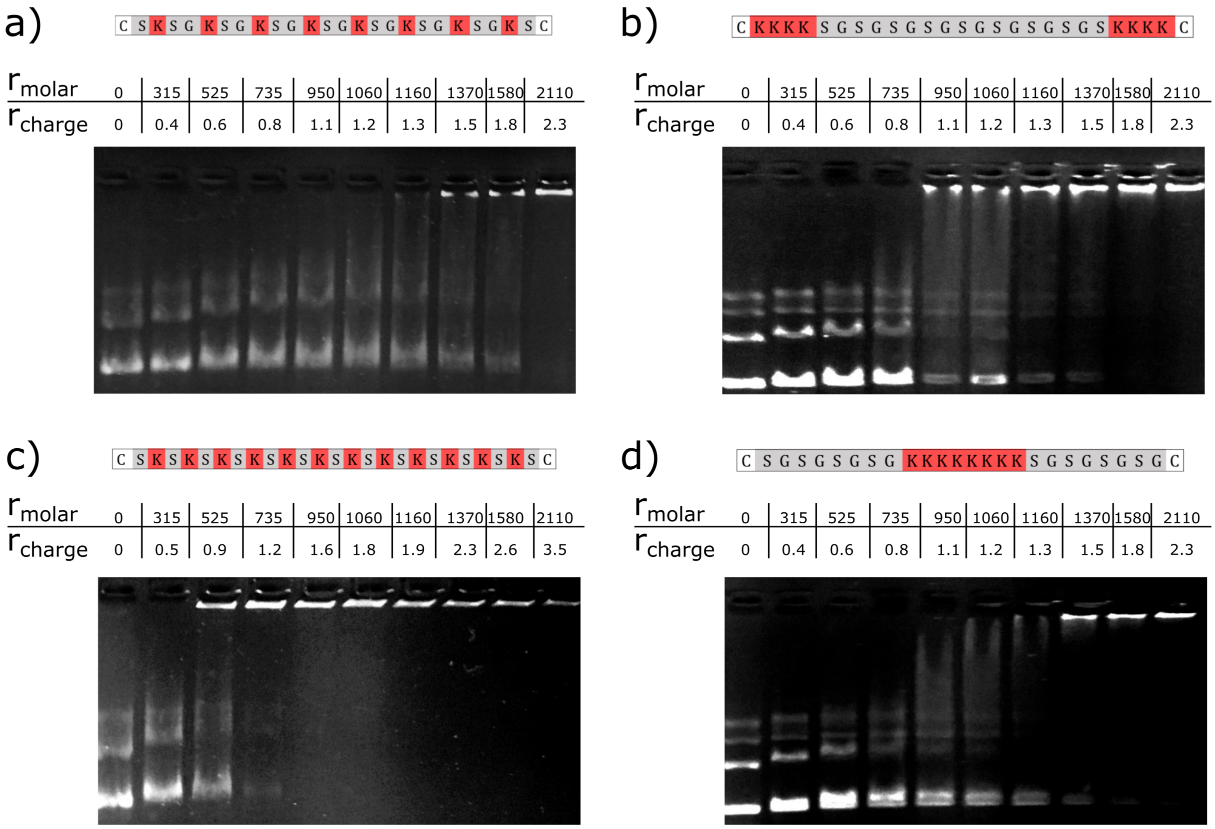

Figure S7: Gel electrophoresis of 10 µg/mL plasmid DNA with increasing concentrations of positively charged peptides. a) $(00P)_8$, b) $P_4 0_{17} P_4$, c)$(0P)_{12}0$ and d) $0_8 P_8 0_8$.

tional cost of dealing with the long-range electrostatic interactions. The rejection of Monte Carlo translation particle moves due to the presence of the spherical simulation cell was lower than 0.5%

Radius of gyration of DNA and peptide tails

fig. S8 shows the normalized rms R_g of the DNA in systems with G2, G2 with one or two tails and tails alone. fig. S9 shows the rms R_g of the tails either conjugated to G2 or not, and in the presence or absence of DNA.

Snapshots

Snapshots of simulations with G2 dendrimers conjugated with a single peptide tail are shown in fig. S10. Here, one negatively charged tail does not prevent the G2 conjugate to bind to the DNA.

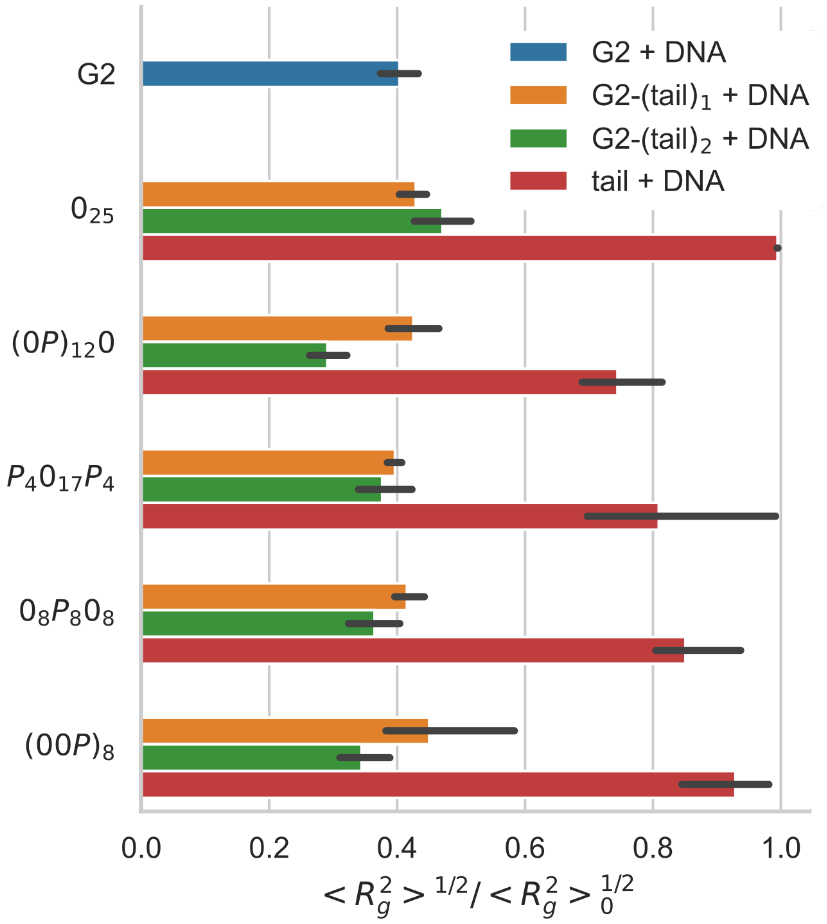

Figure S8: Rms R_g of the DNA in the presence of different vectors (indicated in the plot) normalized by the rms R_g of the DNA alone. All system have $r_{\text{charge}} \approx 1$.

Snapshots of G2 dendrimers and G2 conjugated with neutral, positively charged and negatively charged peptides can be seen in fig. S11. Positively charged tails extend out from the dendrimers while negatively charged tails wrap around the G2.

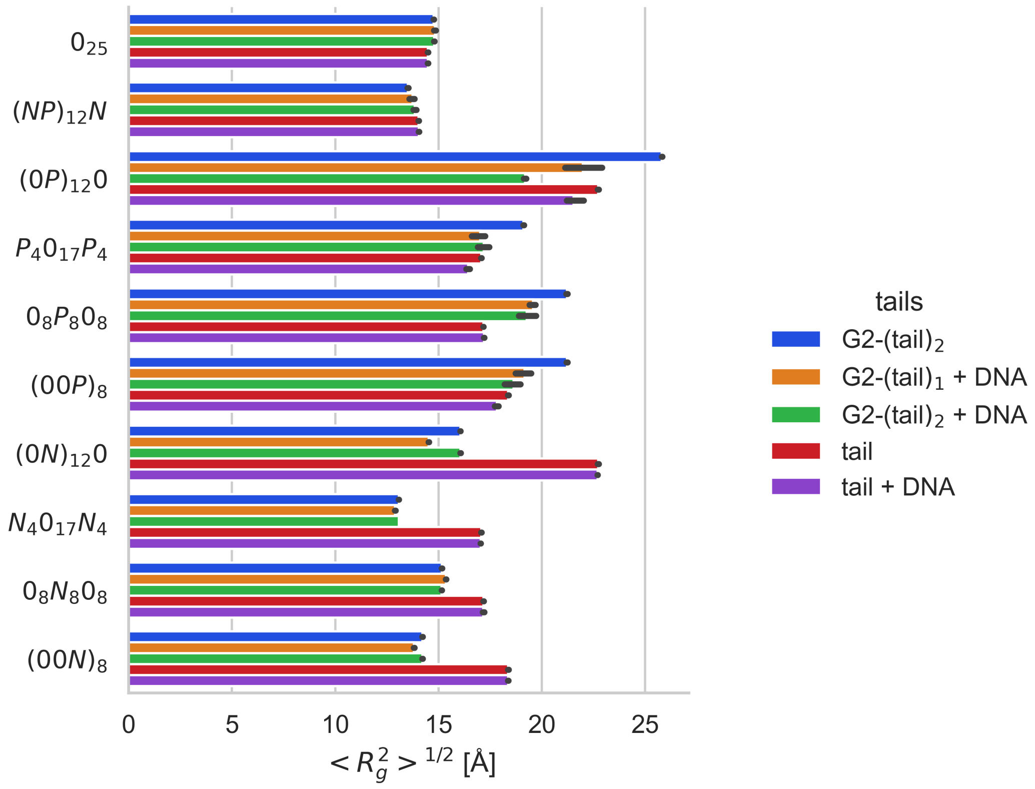

Figure S9: rms R_g of the peptide tails for systems with $r_{\text{charge}} \approx 1$ (with G2 with one or two tails or peptides alone) and G2 conjugates with two tails and peptides alone without DNA.

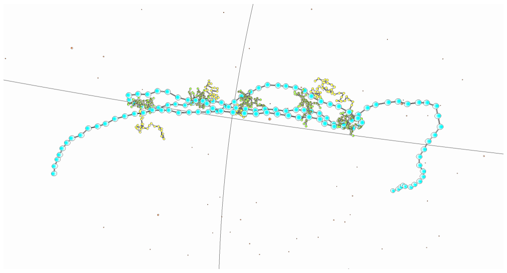

(a) 0_{25}

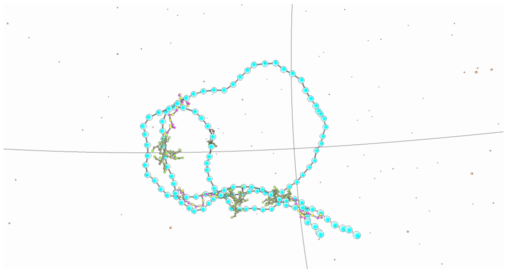

(b) $(0P)_{12}0$

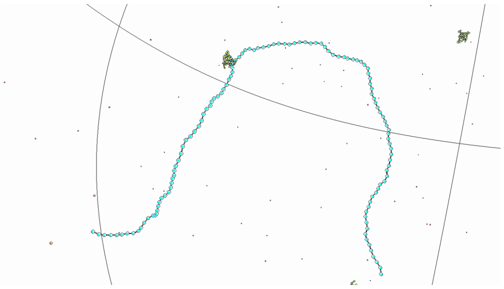

(c) $(0N)_{12}0$

Figure S10: Snapshots of systems include a DNA molecule and G2 with one attached tail. a) neutral tails b) positively charged tails, and c) negatively charged tails.

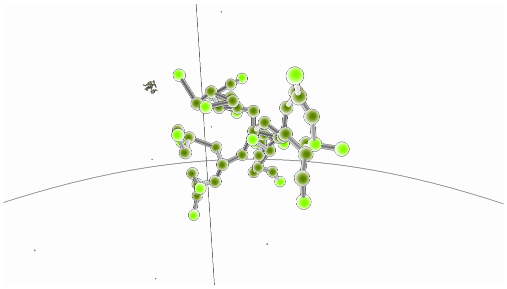

(a) G2 alone

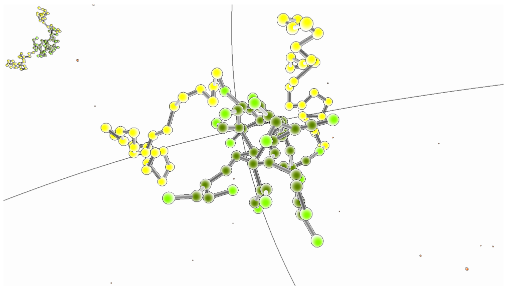

(b) G2-0_{25}

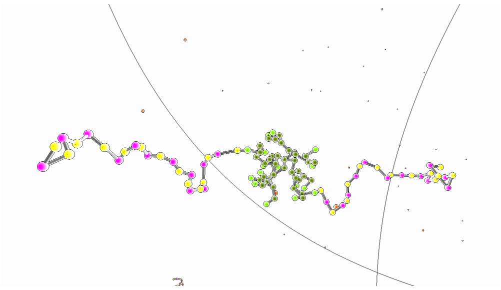

(c) G2-$(0P)_{12}0$

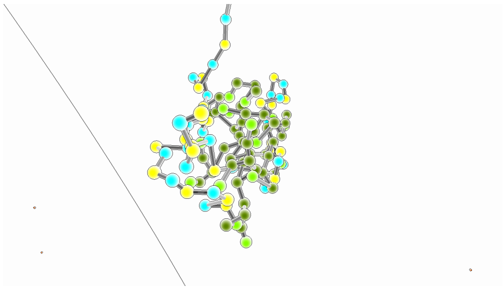

(d) G2-$(0N)_{12}0$

Figure S11: Snapshots of systems with PAMAM alone (a) or with two attached tails (b-d), without DNA present. PAMAM in green, neutral peptide monomers in yellow, positively charged peptide monomers are pink and negatively charged peptide monomers in light blue.
